# Biologic and small-molecule therapies for inflammatory bowel disease: mechanisms, comparative evidence and the emerging pipeline

**DOI:** 10.3389/fphar.2026.1948258

**Published:** 2026-10-07

**Authors:** Longliang Wu, Cuiping Yang, Jianjiong Wu

**Affiliations:** 1 Shanghai Longliang Yuanjian Medical Technology Co., Ltd., Shanghai, China; 2 Professional Committee of High-Quality Multidisciplinary Team Care for Inflammatory Bowel Disease, Beijing Medical Award Foundation, Beijing, China; 3 Department of Gastroenterology, Ruijin Hospital, Shanghai Jiao Tong University School of Medicine, Shanghai, China; 4 Department of Gastroenterology, Shanghai General Hospital, Shanghai Jiao Tong University School of Medicine, Shanghai, China

**Keywords:** biological therapy, Crohn’s disease, inflammatory bowel disease, interleukin-23 inhibitors, Janus kinase inhibitors, small-molecule drugs, treat-to-target, ulcerative colitis

## Abstract

Inflammatory bowel disease (IBD), comprising ulcerative colitis (UC) and Crohn’s disease (CD), is a chronic immune-mediated disorder of rising global prevalence, with newly industrialised nations now entering the phase of most rapid growth. Its therapeutic landscape has transformed since 2020. Three selective interleukin (IL)-23p19 antagonists have been approved for both UC and CD; two sphingosine-1-phosphate receptor modulators and a second-generation Janus kinase (JAK) inhibitor have entered routine practice; subcutaneous formulations have altered delivery; and the first formal head-to-head trials, together with placebo-controlled trials incorporating an active reference arm, have begun to replace indirect comparison with direct evidence. In parallel, the ORAL Surveillance trial prompted a class-wide boxed warning that recalibrated, but did not eliminate, the role of JAK inhibitors. Three further strategies have generated late-phase data: anti-TL1A antibodies, which target a pathway not previously addressed pharmacologically in IBD; the miR-124 enhancer obefazimod; and the oral IL-23 receptor antagonist peptide icotrokinra. Several of these results are available only from conference presentations or company announcements and await peer review. Despite this expansion, induction remission rates remain clustered between roughly 15% and 50%, and endoscopic remission rates lower still. This review synthesises the mechanistic basis, pivotal evidence, comparative effectiveness, safety and regulatory status of approved and investigational therapies for IBD, and how treat-to-target principles, early advanced therapy and emerging biomarkers are reshaping positioning. It also covers two areas comparable reviews omit: acute severe ulcerative colitis, where advanced therapy is least well evidenced, and positioning in newly industrialised nations, where tuberculosis and hepatitis B endemicity and reimbursement, not efficacy alone, often determine the choice of mechanism.

## 1 Introduction

Inflammatory bowel disease (IBD) is an umbrella term for chronic, relapsing and remitting inflammatory disorders of the gastrointestinal tract, principally ulcerative colitis (UC) and Crohn’s disease (CD) (Rubin et al., 2025b). UC is characterised by diffuse, continuous, non-specific mucosal inflammation extending proximally from the rectum, frequently producing erosion and ulceration confined to the mucosa and submucosa. CD, by contrast, is characterised by discontinuous, transmural inflammation that may affect any segment of the gastrointestinal tract and is complicated by stricture and fistula formation; epithelioid granulomas are supportive of the diagnosis when present but are identified in only a minority of cases and are not required (Lichtenstein et al., 2018). Both present with abdominal pain, diarrhoea, rectal bleeding and systemic features such as weight loss, fever and fatigue, and both are associated with extraintestinal manifestations involving the skin, eyes, joints and hepatobiliary system.

The epidemiology of IBD has entered a new phase. Kaplan and Windsor (2021) describe four epidemiological stages through which populations pass: emergence, acceleration in incidence, compounding prevalence and prevalence equilibrium. Early-industrialised regions of North America, Europe and Oceania have entered the compounding-prevalence stage, in which incidence stabilises while prevalence continues to rise as incident cases accumulate, such that more than 1% of these populations are projected to be living with IBD within the next decade (Kaplan, 2015; Kaplan and Windsor, 2025). A claims-based analysis estimated the prevalence of diagnosed IBD in the United States at approximately 0.7%, corresponding to 2.4 million people (Lewis et al., 2023). Newly industrialised nations are following the same trajectory approximately three decades behind (Ng et al., 2017): incidence in China rose steeply between 1990 and 2021, and modelling suggests that China will transition from accelerating incidence to compounding prevalence around 2030 (Shao et al., 2022). This matters therapeutically as well as for service planning, because the populations in which the advanced-therapy armamentarium is expanding fastest are not those in which it was developed. Because IBD is incurable, is diagnosed most often in adolescence and early adulthood, and carries lifelong risks of hospitalisation, surgery and disability, this demographic shift will impose a substantial and sustained burden on health systems.

Historically, pharmacotherapy for IBD rested on four pillars: 5-aminosalicylates, corticosteroids, antibiotics and conventional immunomodulators such as thiopurines and methotrexate. These agents are cheap and familiar but are limited by modest efficacy in moderate-to-severe disease, cumulative toxicity and—in the case of corticosteroids—an inability to maintain remission or heal mucosa. The introduction of infliximab for CD in 1998 inaugurated the era of advanced therapy, defined here as biologic agents and targeted small molecules directed at specific immune pathways. In the quarter-century since, the number of mechanistically distinct advanced therapies has grown from one to more than a dozen (Table 1; Figure 1), and the pace of approval has accelerated sharply: between 2021 and 2026 alone, regulators approved two S1P receptor modulators, a second JAK inhibitor and three IL-23p19 antagonists, several of which now carry indications for both UC and CD.

**TABLE 1:** Biologic and small-molecule therapies approved for inflammatory bowel disease in the United States, July 2026.

Agent	Molecular target	Induction	Maintenance	UC	CD
Infliximab (and biosimilars)	TNF-α	5 mg/kg IV, weeks 0, 2, 6	5 mg/kg IV every 8 weeks	2005	1998
Infliximab, subcutaneous (CT-P13 SC)	TNF-α	IV induction	120 mg SC every 2 weeks	2023	2023
Adalimumab (and biosimilars)	TNF-α	160/80 mg SC	40 mg SC every 2 weeks	2012	2007
Golimumab	TNF-α	200/100 mg SC	50–100 mg SC every 4 weeks	2013	—
Certolizumab pegol	TNF-α (Fab’ fragment)	400 mg SC, weeks 0, 2, 4	400 mg SC every 4 weeks	—	2008
Natalizumab	α4 integrin subunit	300 mg IV	300 mg IV every 4 weeks	—	2008
Vedolizumab	α4β7 integrin	300 mg IV, weeks 0, 2, 6	300 mg IV every 8 weeks or 108 mg SC every 2 weeks	2014 (SC 2023)	2014 (SC 2024)
Ustekinumab (and biosimilars)	IL-12/23 p40 subunit	Weight-based IV	90 mg SC every 8 weeks	2019	2016
Risankizumab	IL-23 p19 subunit	600 mg (CD) or 1,200 mg (UC) IV	180 or 360 mg SC every 8 weeks	2024	2022
Mirikizumab	IL-23 p19 subunit	300 mg IV every 4 weeks	200 mg SC every 4 weeks	2023	2025
Guselkumab	IL-23 p19 subunit; binds CD64	200 mg IV or 400 mg SC every 4 weeks	100 mg SC every 8 weeks or 200 mg SC every 4 weeks	2024	2025
Tofacitinib	JAK1 and JAK3	10 mg orally twice daily	5–10 mg orally twice daily	2018	—
Upadacitinib	JAK1 (selective)	45 mg orally once daily	15 or 30 mg orally once daily	2022	2023
Ozanimod	S1P_1_ and S1P_5_	Oral 7-day titration	0.92 mg orally once daily	2021	—
Etrasimod	S1P_1_, S1P_4_ and S1P_5_	2 mg orally once daily	2 mg orally once daily	2023	—

Years denote the first United States Food and Drug Administration approval for each indication. Agents approved elsewhere but not in the United States are not listed: filgotinib (JAK1) is approved for ulcerative colitis in the European Union; carotegrast methyl, an oral α4 integrin antagonist, is approved in Japan for ulcerative colitis with treatment limited to 6 months; and ivarmacitinib (JAK1), developed in China, is approved there for ankylosing spondylitis, rheumatoid arthritis, atopic dermatitis and severe alopecia areata, with the ulcerative colitis programme in phase 3. CD, Crohn’s disease; IL, interleukin; IV, intravenous; JAK, Janus kinase; S1P, sphingosine-1-phosphate; SC, subcutaneous; TNF, tumour necrosis factor; UC, ulcerative colitis.

**FIGURE 1:**
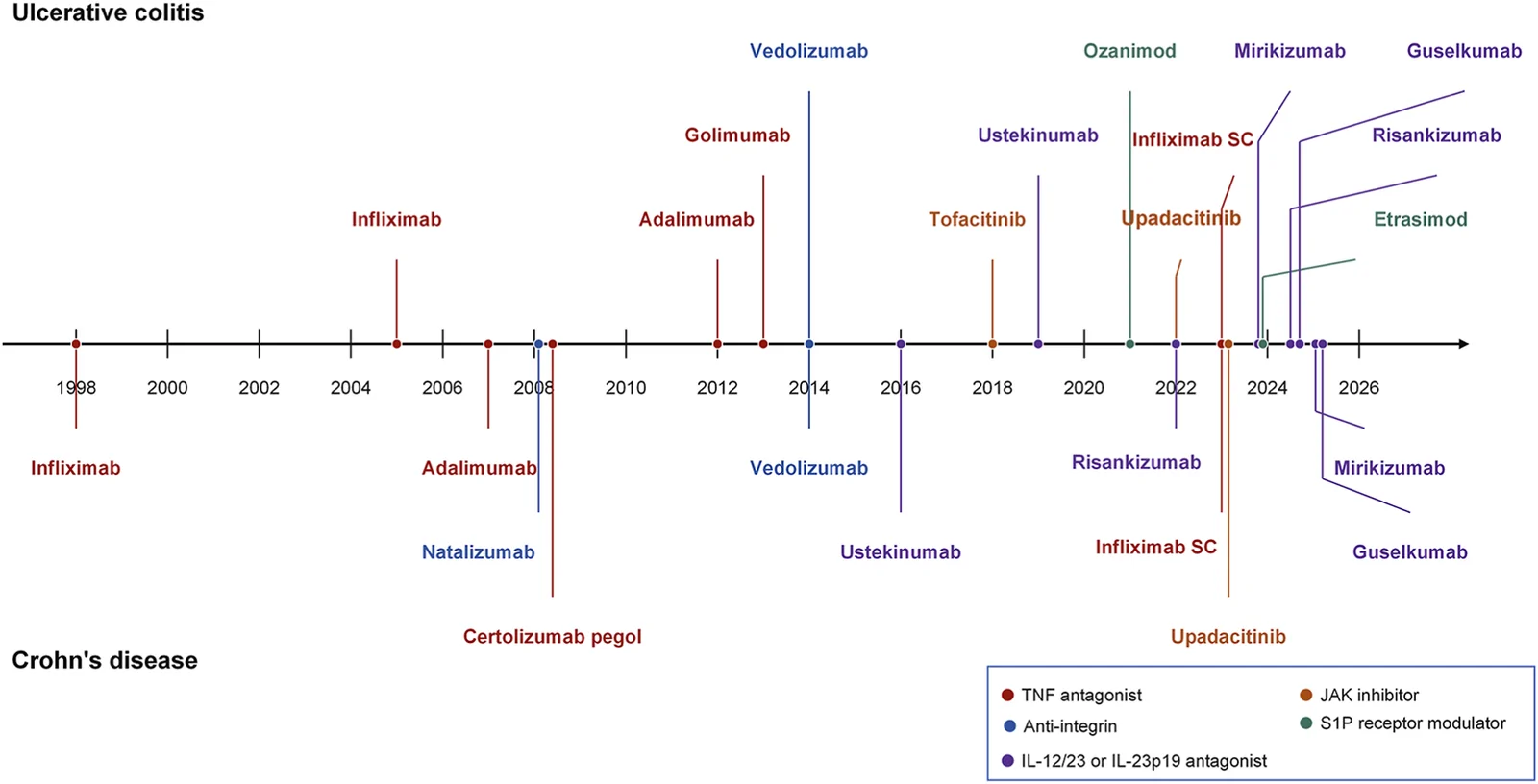
Timeline of United States Food and Drug Administration approvals of advanced therapies for ulcerative colitis (above the axis) and Crohn’s disease (below the axis), 1998–2026, coloured by mechanistic class. The clustering of approvals after 2021 illustrates the rapid recent expansion of the therapeutic armamentarium, and in particular the emergence of the interleukin-23p19 antagonist class with indications in both diseases.

This expansion has created a new clinical problem. Where the question was once whether an advanced therapy should be used, it is now which agent should be used first, how response should be measured, when and how to switch, and how the competing considerations of efficacy, safety, route of administration, monitoring burden and cost should be weighed for an individual patient. This review therefore has four aims: to summarise the mechanistic rationale and pivotal evidence for each therapeutic class; to synthesise the comparative effectiveness data—including the head-to-head trials that have only recently become available—that now inform positioning; to describe the late-stage pipeline, particularly the anti-TL1A antibodies, obefazimod and oral IL-23 receptor antagonists, the first of which addresses a pathway not previously targeted pharmacologically in IBD, the second of which acts by enhancing an endogenous regulatory pathway rather than by blocking a cytokine, and the third of which represents a novel route of administration for an established pathway; and to examine two settings in which positioning departs from the guideline default—acute severe ulcerative colitis, and the newly industrialised nations in which endemic infection and reimbursement policy constrain the choice of mechanism.

## 2 Literature search strategy

This is a narrative review informed by a structured search. PubMed/MEDLINE, Embase and the Cochrane Central Register of Controlled Trials were searched from inception to July 2026 using combinations of the terms “inflammatory bowel disease,” “ulcerative colitis,” “Crohn disease,” “biological therapy,” “tumor necrosis factor-alpha,” “interleukin-23,” “integrin,” “Janus kinase,” “sphingosine-1-phosphate,” “TL1A” and the generic name of each agent discussed. ClinicalTrials.gov was searched for ongoing phase 2 and 3 programmes, and regulatory status was verified against announcements from the US Food and Drug Administration, the European Medicines Agency, the Japanese Pharmaceuticals and Medical Devices Agency and the Chinese National Medical Products Administration. Priority was given to randomised controlled trials, head-to-head comparisons, network meta-analyses and society guidance. Where data derive from conference abstracts or company announcements that have not completed peer review, this is stated explicitly wherever such data are cited.

## 3 Immunological rationale for targeted therapy

The pathogenesis of IBD reflects a dysregulated mucosal immune response to the intestinal microbiota in a genetically susceptible host with a compromised epithelial barrier. Three broad nodes in this network have proved therapeutically tractable, and each defines a class of advanced therapy: the mucosal cytokine network and the epithelial barrier it acts upon (Figure 2B), leukocyte trafficking to the gut (Figure 2A), and intracellular signal transduction (Figure 2C). They are discussed below in that order.

**FIGURE 2:**
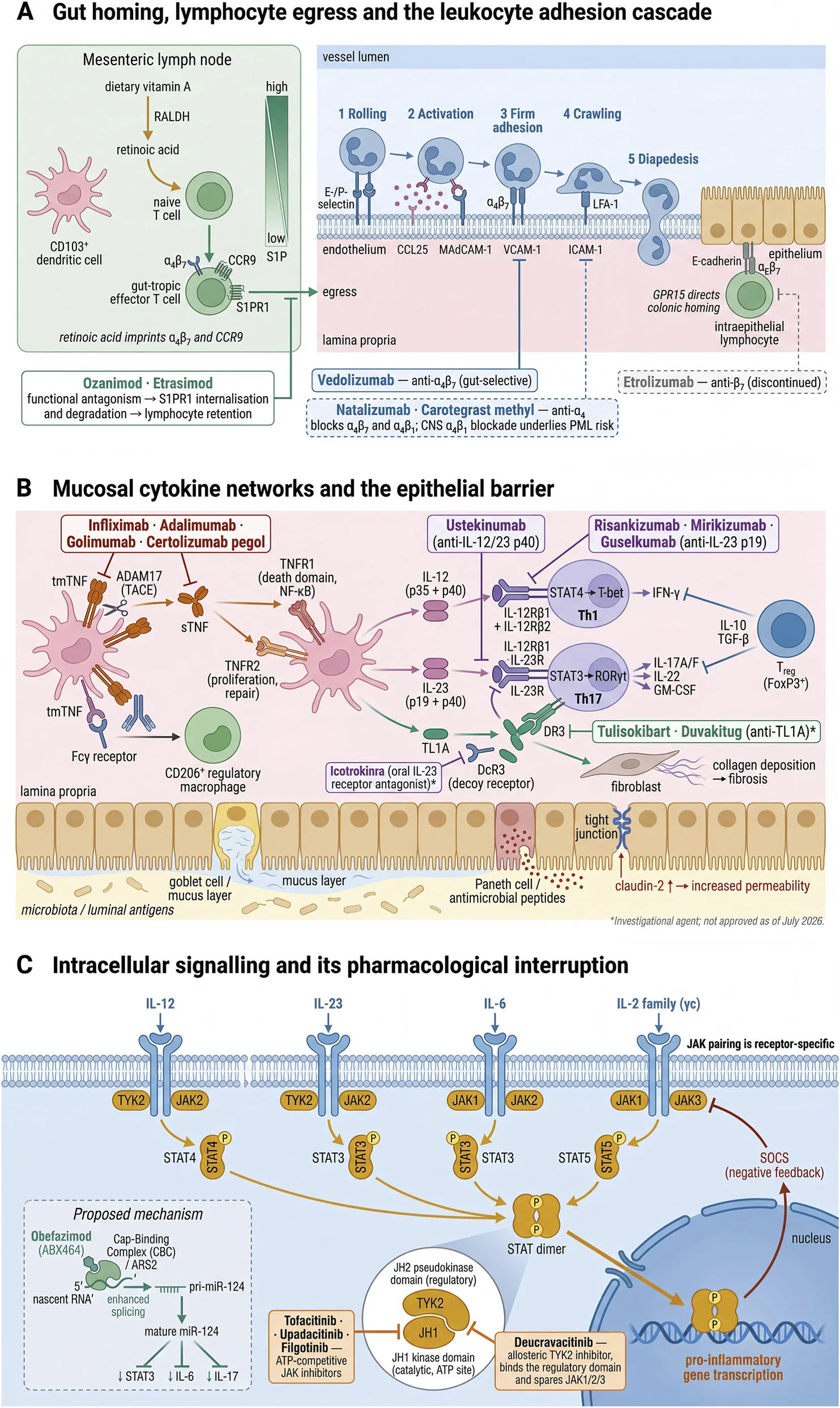
Molecular targets of approved and late-stage investigational therapies in inflammatory bowel disease. **(A)** Gut homing, lymphocyte egress and the leukocyte adhesion cascade. In the mesenteric lymph node, CD103-positive dendritic cells metabolise dietary vitamin A to retinoic acid through retinaldehyde dehydrogenase (RALDH), imprinting α4β7 and CCR9 on naive T cells and conferring gut tropism. Egress follows the sphingosine-1-phosphate (S1P) gradient and requires S1P receptor 1 (S1PR1); ozanimod and etrasimod act as functional antagonists that drive S1PR1 internalisation and degradation, sequestering lymphocytes within lymphoid tissue. At the intestinal postcapillary venule the adhesion cascade proceeds in five steps: selectin-mediated tethering and rolling; chemokine-triggered inside-out integrin activation by CCL25; firm adhesion through α4β7–MAdCAM-1, with contributions from α4β1–VCAM-1 and LFA-1–ICAM-1; crawling; and diapedesis. Vedolizumab blocks α4β7 selectively, whereas natalizumab and carotegrast methyl block the shared α4 subunit and therefore also α4β1, the interaction governing lymphocyte entry into the central nervous system that underlies the risk of progressive multifocal leukoencephalopathy. Within the epithelium, αEβ7–E-cadherin binding retains intraepithelial lymphocytes; etrolizumab, directed at the shared β7 subunit, did not complete development. **(B)** Mucosal cytokine networks and the epithelial barrier. Transmembrane TNF is cleaved by ADAM17 (TNF-α-converting enzyme) to soluble TNF; TNFR1 carries a death domain and drives nuclear factor-κB-dependent inflammation, whereas TNFR2 mediates proliferation and repair. Whole IgG1 antibodies additionally engage Fcγ receptors and induce CD206-positive regulatory macrophages, mechanisms unavailable to the Fc-deficient fragment certolizumab pegol. IL-12 (p35 + p40) signals through IL-12Rβ1 and IL-12Rβ2 to STAT4 and T-bet, driving type 1 helper T cell differentiation and interferon-γ; IL-23 (p19 + p40) signals through IL-12Rβ1 and IL-23R to STAT3 and RORγt, driving type 17 responses and IL-17A/F, IL-22 and granulocyte-macrophage colony-stimulating factor. Ustekinumab neutralises the shared p40 subunit and therefore both cytokines, whereas risankizumab, mirikizumab and guselkumab bind p19 selectively and icotrokinra antagonises the IL-23 receptor. TL1A engages death receptor 3 on effector T cells and on fibroblasts, where it promotes collagen deposition and fibrosis; the decoy receptor DcR3 sequesters TL1A. Regulatory T cells restrain effector responses through IL-10 and transforming growth factor-β. The epithelial barrier comprises goblet cells and the mucus layer, Paneth cells and their antimicrobial peptides, and tight junctions in which upregulated claudin-2 increases paracellular permeability. **(C)** Intracellular signalling and its pharmacological interruption. Janus kinase pairing is receptor-specific: IL-12 and IL-23 signal through TYK2 and JAK2 to STAT4 and STAT3 respectively, IL-6 through JAK1 and JAK2 to STAT3, and the IL-2 family through JAK1 and JAK3 to STAT5. Phosphorylated STATs dimerise, translocate to the nucleus and drive pro-inflammatory gene transcription, which is restrained by suppressor of cytokine signalling-mediated negative feedback. Tofacitinib, upadacitinib and filgotinib compete with ATP at the JH1 catalytic domain, whereas deucravacitinib binds the regulatory JH2 pseudokinase domain of TYK2 allosterically and spares JAK1, JAK2 and JAK3. In the proposed mechanism of obefazimod, the compound binds the cap-binding complex and ARS2 at the 5′ cap of nascent RNA, enhancing splicing of pri-miR-124 to mature miR-124 and thereby suppressing STAT3, IL-6 and IL-17; this pathway is shown as proposed because the causal chain has not been established *in vivo*. Asterisks denote investigational agents not approved as of July 2026.

The first node is effector cytokine signalling. Tumour necrosis factor (TNF)-α, produced principally by macrophages, dendritic cells and T cells, activates nuclear factor-κB and mitogen-activated protein kinase cascades and initiates caspase-dependent pathways, driving epithelial apoptosis, matrix metalloproteinase release, angiogenesis and further leukocyte recruitment (Bradley, 2008). The IL-12 and IL-23 heterodimers, which share a common p40 subunit, direct the differentiation and maintenance of type 1 and type 17 helper T cell responses respectively; IL-23 in particular sustains a pathogenic, granulocyte-macrophage colony-stimulating factor-producing T helper 17 phenotype in the inflamed intestine (Teng et al., 2015). TNF-like ligand 1A (TL1A), a more recently characterised TNF superfamily member signalling through death receptor 3, amplifies multiple effector lineages and additionally drives intestinal fibrosis, making it an attractive target in stricturing disease.

The second node is leukocyte trafficking. Lymphocytes primed in gut-associated lymphoid tissue acquire gut-homing receptors and must complete two steps to reach the inflamed mucosa (Figure 2A): egress from lymphoid organs, which depends on the S1P receptor 1 (S1P1) gradient, and adhesion to and transmigration across intestinal endothelium, which depends on the interaction between the α4β7 integrin and mucosal addressin cell adhesion molecule-1 (MAdCAM-1) expressed on high endothelial venules of the intestinal vasculature (Hynes, 1987; Erle et al., 1994). A further integrin, αEβ7, binds epithelial E-cadherin and retains lymphocytes within the epithelial compartment. Because α4β7–MAdCAM-1 signalling is largely restricted to the gut, its blockade offers organ-selective immunosuppression, which underlies the favourable systemic safety profile of this class.

The third node is intracellular signal transduction. Many of the cytokines implicated in IBD, including IL-6, IL-9, IL-21, IL-23 and the interferons, signal through receptors coupled to Janus kinases (JAK1, JAK2, JAK3 and tyrosine kinase 2), which phosphorylate signal transducer and activator of transcription (STAT) proteins and thereby control pro-inflammatory gene transcription. Small-molecule JAK inhibitors interrupt several cytokine pathways simultaneously, which explains both their rapid onset of action and their broader safety liabilities. A more recent addition to this node is the modulation of microRNA biology: obefazimod selectively upregulates miR-124, an endogenous brake on STAT3, IL-6 and IL-17 signalling.

Interruption of intracellular signalling may also be barrier-protective in its own right. Several JAK-dependent cytokines, notably interferon-γ, IL-6 and IL-13, impair tight-junction integrity and increase paracellular permeability in intestinal epithelial cells, and JAK inhibition has been shown in human epithelial monolayers and colonoids, and in murine models, to prevent or reverse cytokine-induced barrier dysfunction (Sayoc-Becerra et al., 2020; Spalinger et al., 2021). This evidence is preclinical, and barrier endpoints have not been assessed in clinical trials, but it suggests that cytokine-directed and signalling-directed strategies may converge on the same epithelial target. These nodes are not independent, which may explain why no monotherapy has exceeded a remission ceiling of roughly half of treated patients in placebo-controlled trials of moderate-to-severe disease and why combination approaches are now being tested formally.

## 4 Tumour necrosis factor antagonists

### 4.1 Agents and mechanism of action

Five TNF antagonists have been developed for immune-mediated inflammatory disease, but only four are effective in IBD. Infliximab is a chimeric mouse–human immunoglobulin (Ig) G1 monoclonal antibody; adalimumab and golimumab are fully human IgG1 antibodies; certolizumab pegol is a PEGylated Fab’ fragment lacking an Fc region; and etanercept is a soluble TNF receptor 2–IgG1 Fc fusion protein.

Etanercept neutralises soluble TNF as effectively as the monoclonal antibodies but is ineffective in CD (Figure 2B) (Levin et al., 2016). This dissociation established that TNF neutralisation alone is insufficient. The whole antibodies bind transmembrane TNF on activated monocytes and T cells and thereby deliver outside-to-inside reverse signalling that induces apoptosis, engage Fcγ receptors on macrophages to promote antibody-dependent cellular cytotoxicity, and drive the differentiation of CD206-positive regulatory macrophages that suppress T cell proliferation and promote mucosal healing (Levin et al., 2016). Certolizumab pegol, which lacks an Fc region, occupies an intermediate position and has generally shown more modest efficacy than the whole antibodies, an observation that has been attributed to the absence of Fc-dependent mechanisms although the attribution remains debated (Sandborn et al., 2011). These mechanistic distinctions illustrate why molecular architecture, and not target alone, determines clinical outcome in IBD.

### 4.2 Efficacy

Infliximab remains among the most effective agents available for both diseases. In the ACT 1 trial, clinical remission at week 8 was achieved by 38.8% of patients receiving infliximab 5 mg/kg compared with 14.9% receiving placebo, and by week 54 the corresponding figures were 34.7% and 16.5% (Rutgeerts et al., 2005). Adalimumab and golimumab are effective but with smaller effect sizes in UC; in ULTRA 2, week 8 remission occurred in 16.5% versus 9.3% with placebo (Sandborn et al., 2012). Infliximab and adalimumab are both effective in perianal fistulising CD, and infliximab reduces hospitalisation and surgery (Costa et al., 2013). For postoperative recurrence, treatment guided by early colonoscopy reduced endoscopic recurrence at 18 months compared with standard care (49% versus 67%) (De Cruz et al., 2015). Whereas infliximab reduced endoscopic but not clinical recurrence after ileocolonic resection (Regueiro et al., 2016). Against this background, population-based cohorts show a substantial decline in surgical rates in both diseases across the biologic era (Tsai et al., 2021). Figure 3 places these effect sizes alongside those of newer agents, and Tables 2 and 3 summarise the pivotal induction and maintenance results for ulcerative colitis and Crohn’s disease respectively.

**FIGURE 3:**
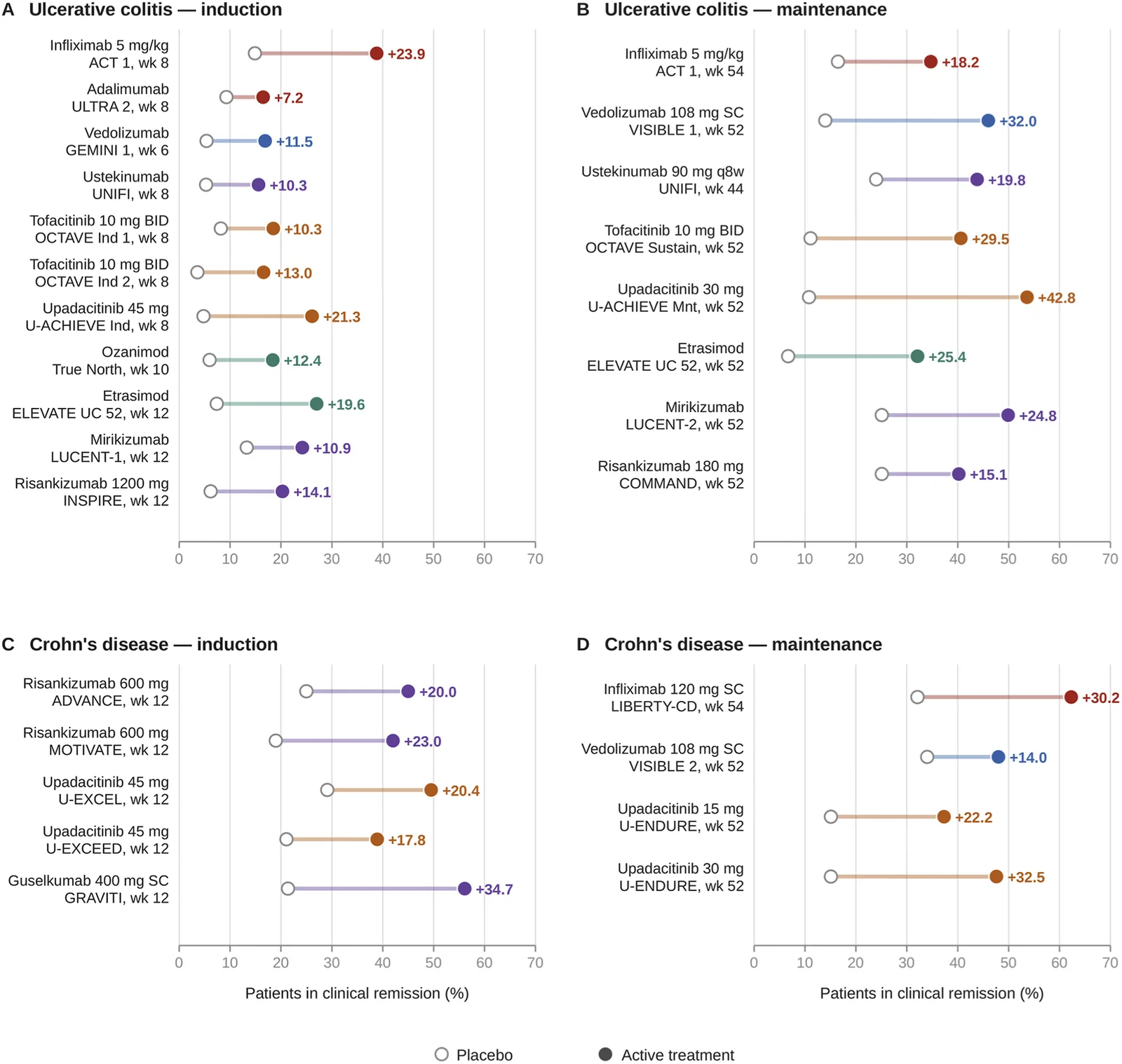
Clinical remission rates with active treatment (filled circles) and placebo (open circles) in pivotal trials of advanced therapies in ulcerative colitis **(A)** induction; **(B)** maintenance and Crohn’s disease **(C)** induction; **(D)** maintenance. Values adjacent to each pair indicate the absolute placebo-adjusted difference in percentage points. Definitions of clinical remission, timing of assessment, prior treatment exposure and trial design differ substantially between studies—notably between treat-through and randomised-withdrawal maintenance designs—so these data illustrate the magnitude and consistency of treatment effects rather than supporting direct cross-trial comparison. Trial abbreviations and time points are given beside each agent.

**TABLE 2:** Clinical remission in pivotal randomised trials of advanced therapies in ulcerative colitis.

Agent (dose)	Trial	Endpoint	Active (%)	Placebo (%)	Difference (pp)
Induction					
Infliximab 5 mg/kg	ACT 1	Clinical remission, week 8	38.8	14.9	23.9
Adalimumab	ULTRA 2	Clinical remission, week 8	16.5	9.3	7.2
Vedolizumab 300 mg	GEMINI 1	Clinical remission, week 6	16.9	5.4	11.5
Ustekinumab (IV induction)	UNIFI	Clinical remission, week 8	15.6	5.3	10.3
Tofacitinib 10 mg twice daily	OCTAVE Induction	Remission, week 8	18.5	8.2	10.3
Tofacitinib 10 mg twice daily	OCTAVE Induction	Remission, week 8	16.6	3.6	13.0
Upadacitinib 45 mg once daily	U‐ACHIEVE Induction	Clinical remission, week 8	26.1	4.8	21.3
Ozanimod 0.92 mg once daily	True North	Clinical remission, week 10	18.4	6.0	12.4
Etrasimod 2 mg once daily	ELEVATE UC 52	Clinical remission, week 12	27.0	7.4	19.6
Mirikizumab 300 mg IV	LUCENT-1	Clinical remission, week 12	24.2	13.3	10.9
Risankizumab 1,200 mg IV	INSPIRE	Clinical remission, week 12	20.3	6.2	14.1
Maintenance					
Infliximab 5 mg/kg	ACT 1	Clinical remission, week 54	34.7	16.5	18.2
Vedolizumab 108 mg SC	VISIBLE 1	Clinical remission, week 52	46.0	14.0	32.0
Ustekinumab 90 mg every 8 weeks	UNIFI	Clinical remission, week 44	43.8	24.0	19.8
Tofacitinib 10 mg twice daily	OCTAVE Sustain	Remission, week 52	40.6	11.1	29.5
Upadacitinib 30 mg once daily	U-ACHIEVE Maintenance	Clinical remission, week 52	53.6	10.8	42.8
Etrasimod 2 mg once daily	ELEVATE UC 52	Clinical remission, week 52	32.1	6.7	25.4
Mirikizumab 200 mg SC	LUCENT-2	Clinical remission, week 52	49.9	25.1	24.8
Risankizumab 180 mg SC	COMMAND	Clinical remission, week 52	40.2	25.1	15.1

Definitions of clinical remission, timing of assessment, permitted prior therapy and trial design differ between studies. Maintenance results from randomised-withdrawal designs (UNIFI, OCTAVE Sustain, U‐ACHIEVE Maintenance, COMMAND, VISIBLE 1) are restricted to induction responders and are not directly comparable with treat-through designs (ACT 1, ELEVATE UC 52). These data should therefore not be used for direct cross-trial comparison. IV, intravenous; pp, percentage points; SC, subcutaneous.

**TABLE 3:** Clinical remission in pivotal randomised trials of advanced therapies in Crohn’s disease.

Agent (dose)	Trial	Endpoint	Active (%)	Placebo (%)	Difference (pp)
Induction					
Risankizumab 600 mg IV	ADVANCE	CDAI clinical remission, week 12	45	25	20
Risankizumab 600 mg IV	MOTIVATE	CDAI clinical remission, week 12	42	19	23
Upadacitinib 45 mg once daily	U-EXCEL	Clinical remission, week 12	49.5	29.1	20.4
Upadacitinib 45 mg once daily	U-EXCEED	Clinical remission, week 12	38.9	21.1	17.8
Guselkumab 400 mg SC	GRAVITI	Clinical remission, week 12	56.1	21.4	34.7
Maintenance					
Infliximab 120 mg SC	LIBERTY-CD	Clinical remission, week 54	62.3	32.1	30.2
Vedolizumab 108 mg SC	VISIBLE 2	Clinical remission, week 52	48	34	14
Upadacitinib 15 mg once daily	U-ENDURE	Clinical remission, week 52	37.3	15.1	22.2
Upadacitinib 30 mg once daily	U-ENDURE	Clinical remission, week 52	47.6	15.1	32.5

MOTIVATE enrolled only patients with prior biologic failure, whereas ADVANCE permitted prior conventional or biologic therapy, which accounts for the lower placebo rate in MOTIVATE. ADVANCE and MOTIVATE also assessed endoscopic response as a co-primary endpoint. GRAVITI used a treat-through design with fully subcutaneous induction. As in Table 2, differences in endpoint definition and design preclude direct cross-trial comparison. CDAI, Crohn’s Disease Activity Index; IV, intravenous; pp, percentage points; SC, subcutaneous.

Response to a second TNF antagonist after failure of the first depends on why the first was stopped. A meta-analysis of 37 studies found an overall clinical remission rate of 43% with a second agent, but rates differed substantially according to the reason for discontinuation: 61% when the first agent was stopped for intolerance, 45% after secondary loss of response, and only 30% after primary non-response (Gisbert et al., 2015). This gradient underpins the modern principle that primary non-response to an adequately dosed TNF antagonist should prompt a change of mechanism rather than a change of molecule within the class.

### 4.3 Immunogenicity, therapeutic drug monitoring and combination therapy

The pharmacological rationale for monitoring rests on the pharmacokinetics of monoclonal antibodies. Immunoglobulin G antibodies are eliminated principally by non-specific proteolytic catabolism, with a variable contribution from target-mediated drug disposition, and are protected from degradation by neonatal Fc receptor (FcRn)-mediated recycling; the TNF antagonists have terminal half-lives of the order of one to two weeks, and clearance varies severalfold between patients. Higher body weight, a high inflammatory burden, male sex and the presence of anti-drug antibodies all increase clearance and lower trough concentration, as does hypoalbuminaemia, since albumin and IgG compete for the same FcRn salvage pathway, and in severe colitis the inflamed mucosa itself becomes a route of protein loss, so that drug is lost in the stool at the point of greatest disease activity (see Section 10.5). Because trough concentration correlates with clinical and endoscopic outcome across the TNF antagonists, these sources of variability define an exposure-response relationship, and it is this relationship, rather than any property of the molecule alone, that therapeutic drug monitoring seeks to exploit. Loss of response is common and is frequently driven by immunogenicity. The PANTS study demonstrated that carriage of the HLA‐DQA1*05 allele, present in approximately 40% of people of European ancestry, nearly doubles the hazard of developing anti-drug antibodies (hazard ratio 1.90, 95% confidence interval 1.60–2.25) (Sazonovs et al., 2020). The interaction with drug and co-therapy is striking: the rate of immunogenicity at 1 year ranged from 92% for infliximab monotherapy in carriers to 10% for adalimumab combination therapy in non-carriers (Sazonovs et al., 2020). HLA‐DQA1*05 genotyping is consequently among the first pharmacogenomic tests with a plausible route into routine IBD practice.

Combination therapy with an immunomodulator reduces immunogenicity and improves outcomes, as established by SONIC in CD (Colombel et al., 2010) and UC-SUCCESS in UC (Panaccione et al., 2014). The evidence on therapeutic drug monitoring is best read as distinguishing induction from maintenance. TAXIT found that once all patients had been optimised to a trough of 3–7 μg/mL, continued concentration-based dosing was not superior to clinical dosing for 1-year remission (Vande Casteele et al., 2015), and TAILORIX likewise showed no benefit from proactive escalation in CD (D’Haens et al., 2018). The NOR-DRUM programme addressed both settings: proactive monitoring during induction did not improve 30-week remission (50.5% versus 53.0%; adjusted difference −1.9%, 95% CI −11.5 to 7.9) (Syversen et al., 2021a), whereas proactive monitoring during maintenance improved sustained disease control (73.6% versus 55.9%; adjusted difference 17.6%, 95% CI 9.0–26.2) (Syversen et al., 2021b). These trials nevertheless enrolled six immune-mediated diseases, of which IBD accounted for approximately one-third of participants (137 of 398 in the induction trial and 149 of 458 in the maintenance trial), and were not powered to test disease-specific effects; the investigators noted that the diseases studied have inherent differences and that the findings may not be fully generalisable across groups. The IBD-specific evidence therefore remains mixed, with benefit from proactive monitoring in NOR-DRUM B and in the paediatric PAILOT trial (Assa et al., 2019) but no benefit for the primary endpoints of TAXIT or TAILORIX, and current AGA guidance endorses reactive monitoring to guide management of loss of response while making no recommendation on routine proactive monitoring (Feuerstein et al., 2017). Proactive monitoring during maintenance is therefore promising but not definitively established in IBD; reactive monitoring remains appropriate at apparent treatment failure.

### 4.4 Subcutaneous infliximab and biosimilars

Two developments have changed the practical delivery of TNF antagonism. First, a subcutaneous formulation of infliximab (CT-P13 SC) has been developed and, in the United States, licensed as a distinct product rather than a biosimilar. In LIBERTY-CD, week 54 clinical remission was achieved by 62.3% of patients receiving subcutaneous infliximab compared with 32.1% receiving placebo, with endoscopic response in 51.1% versus 17.9%; LIBERTY-UC showed clinical remission in 43.2% versus 20.8% (Hanauer et al., 2024). The formulation provides more stable drug concentrations than intermittent intravenous dosing and removes the need for infusion-centre attendance (Celltrion USA, 2023).

Second, biosimilar infliximab and adalimumab have achieved wide penetration. NOR-SWITCH demonstrated that switching from originator infliximab to CT-P13 was non-inferior to continued originator treatment (Jørgensen et al., 2017), and subsequent multiple-switching studies have not identified an efficacy or immunogenicity signal. The resulting price competition has re-established TNF antagonists as the most cost-effective first-line advanced therapy in many health systems—a consideration that now weighs explicitly in positioning decisions.

### 4.5 Safety

The safety profile of this class is well characterised (Table 4). Serious infection is the principal risk; reactivation of latent tuberculosis mandates screening before initiation, and infliximab carries a higher granulomatous infection risk than etanercept (Wallis, 2008). Hepatosplenic T-cell lymphoma is a rare but frequently fatal complication reported predominantly in young men receiving combination thiopurine and TNF antagonist therapy, and is the main argument for limiting the duration of combination therapy in that group. Demyelinating events, drug-induced lupus and paradoxical psoriasiform eruptions are recognised but uncommon. TNF antagonists are among the best-characterised advanced therapies in pregnancy and are generally continued, with consideration given to timing of the final dose in the third trimester.

**TABLE 4:** Class-specific safety considerations, pre-treatment screening and monitoring.

Class	Principal safety signals	Before initiation	During treatment
TNF antagonists	Serious and opportunistic infection; reactivation of latent tuberculosis and hepatitis B; hepatosplenic T-cell lymphoma (young men on combination thiopurine therapy); demyelination; drug-induced lupus; paradoxical psoriasis; infusion and injection-site reactions	Interferon-gamma release assay or tuberculin testing and chest radiograph; hepatitis B and C and HIV serology; varicella and measles immunity; update vaccinations; consider HLA-DQA1*05 genotyping	Full blood count and liver biochemistry; drug concentration and anti-drug antibody measurement (proactively during maintenance, reactively at apparent failure); annual dermatological review
Anti-integrin agents (vedolizumab)	Nasopharyngitis; arthralgia; infusion reactions. No consistent excess of serious or opportunistic infection or malignancy; gut selectivity limits systemic immunosuppression	Routine infection screening; exclude active infection	Minimal laboratory monitoring required; assess response at weeks 6–14, allowing for slow onset of action, particularly in Crohn’s disease
Anti-integrin agents (α4, non-selective)	Progressive multifocal leukoencephalopathy due to JC polyomavirus reactivation (natalizumab); hepatotoxicity (carotegrast methyl)	Anti-JC virus antibody serology and index; document prior immunosuppressant exposure	Periodic anti-JC virus serology; vigilance for new neurological symptoms; carotegrast methyl is limited to 6 months of continuous use
IL-12/23 and IL-23p19 antagonists	Nasopharyngitis; injection-site and infusion reactions; rare hypersensitivity. No consistent signal for serious infection, major adverse cardiovascular events or malignancy in controlled trials and registries	Tuberculosis and viral hepatitis screening; update vaccinations	Minimal routine laboratory monitoring; clinical vigilance for infection
JAK inhibitors	Herpes zoster (dose-dependent); serious infection; major adverse cardiovascular events, venous thromboembolism, malignancy and all-cause mortality (class boxed warning derived from a rheumatoid arthritis population); dyslipidaemia; creatine kinase elevation; cytopenias	Cardiovascular and thromboembolic risk assessment; malignancy history; tuberculosis and viral hepatitis screening; recombinant zoster vaccination; lipid profile, full blood count, liver and renal function	Lipids at 8–12 weeks and periodically; full blood count and liver biochemistry; use the lowest effective maintenance dose; caution in patients over 65 years, smokers and those with cardiovascular risk; both tofacitinib and upadacitinib are CYP3A4 substrates, so dose adjustment is required with strong CYP3A4 inhibitors and efficacy may be reduced by strong inducers
S1P receptor modulators	First-dose bradycardia and atrioventricular conduction delay; macular oedema; hepatic transaminase elevation; lymphopenia; hypertension; infection including varicella zoster; teratogenicity	Electrocardiogram; cardiology advice if pre-existing conduction disease; ophthalmological assessment if diabetes, uveitis or retinal disease; liver biochemistry; full blood count; varicella zoster serology and vaccination if seronegative; pregnancy testing and contraception counselling	Observation during first dose where indicated (ozanimod titration); liver biochemistry and full blood count; ophthalmological review if visual symptoms; review of interacting drugs including CYP2C8 and CYP2C9 substrates and agents that slow atrioventricular conduction; for ozanimod, concomitant monoamine oxidase inhibitors and strong BCRP inhibitors should be avoided because active metabolites are affected

This table summarises considerations common to each class; individual product labels should be consulted for definitive guidance. Live vaccines are contraindicated during treatment with all agents listed. IL, interleukin; JAK, Janus kinase; S1P, sphingosine-1-phosphate; TNF, tumour necrosis factor.

## 5 Agents targeting leukocyte trafficking

### 5.1 Natalizumab

Natalizumab, a humanised IgG4 antibody against the α4 integrin subunit, blocks both α4β7 and α4β1 and was the first anti-trafficking agent shown to be effective in CD. Induction in the ENACT programme did not meet its overall week 10 endpoint, although response was significantly higher in the subgroup with elevated C-reactive protein and maintenance was positive (Sandborn et al., 2005); the confirmatory ENCORE trial, which enrolled only patients with objective inflammation, met all prespecified endpoints (week 8 response 58% versus 45%; remission 40% versus 28%) (Targan et al., 2007). Because α4β1–VCAM-1 interaction governs lymphocyte entry into the central nervous system, pan-α4 blockade carries a risk of progressive multifocal leukoencephalopathy from JC polyomavirus reactivation, mandating risk stratification by anti-JC virus antibody status, prior immunosuppressant exposure and treatment duration. With gut-selective alternatives available, natalizumab is now rarely used in IBD.

### 5.2 Vedolizumab

Vedolizumab is a humanised IgG1 antibody that binds the α4β7 heterodimer specifically, blocking its interaction with MAdCAM-1 without affecting α4β1–VCAM-1 signalling. This gut selectivity is the defining feature of the agent and explains a safety profile that is, across long-term follow-up, among the most favourable of any advanced therapy. Efficacy was established in the GEMINI programme for UC (Feagan et al., 2013) and CD (Sandborn et al., 2013); in GEMINI 1, week 6 clinical remission occurred in 16.9% of vedolizumab-treated patients versus 5.4% with placebo.

Two subsequent developments are clinically important. A subcutaneous maintenance formulation was validated in VISIBLE 1, in which week 52 clinical remission was achieved by 46% of patients receiving vedolizumab 108 mg subcutaneously versus 14% receiving placebo (Sandborn et al., 2020a), and in VISIBLE 2 for CD (48% versus 34%) (Vermeire et al., 2022a). Second, vedolizumab is the first agent with randomised evidence in chronic pouchitis: in the EARNEST trial, week 14 remission by modified Pouchitis Disease Activity Index occurred in 31% of treated patients versus 10% with placebo (difference 21 percentage points, 95% CI 5–38) (Travis et al., 2023), which led to approval for chronic antibiotic-refractory pouchitis in the European Union. Clinicians should note that the onset of action is comparatively slow, particularly in CD, and that vedolizumab is best suited to patients in whom a delay of 8–14 weeks to maximal effect is acceptable.

### 5.3 Etrolizumab: a mechanistically informative programme

Etrolizumab targets the β7 subunit and therefore blocks both α4β7-mediated trafficking and αEβ7-mediated epithelial retention—a dual mechanism that was expected to confer additional benefit over vedolizumab. The phase 3 programme was among the largest ever conducted in IBD, comprising HIBISCUS I and II, LAUREL, HICKORY and GARDENIA in UC and BERGAMOT in CD. Results were inconsistent. Induction endpoints were met in HIBISCUS and in the anti-TNF-experienced HICKORY study (Peyrin-Biroulet et al., 2022; Rubin et al., 2022), but the primary maintenance endpoints were missed in all three maintenance trials—LAUREL, GARDENIA and HICKORY—and etrolizumab did not demonstrate superiority to the active anti-TNF comparators (Danese et al., 2022a; Peyrin-Biroulet et al., 2022; Vermeire et al., 2022b). Efficacy signals were seen in Crohn’s disease in BERGAMOT but did not support continuation (Sandborn et al., 2023b). Development in IBD was discontinued. The episode is a caution against assuming that a broader mechanism will translate into greater efficacy, and it underscores the necessity of full phase 3 programmes with maintenance endpoints before mechanistic reasoning is accepted.

### 5.4 Oral anti-integrin therapy

Carotegrast methyl (AJM300) is an orally active small-molecule α4 antagonist with activity against both α4β7 and α4β1. Following a positive phase 2a study in which patients with an inadequate response to prior therapy achieved higher rates of clinical remission and mucosal healing than with placebo (Yoshimura et al., 2015), a phase 3 trial confirmed efficacy in moderately active UC (Matsuoka et al., 2022), and the agent was approved in Japan in March 2022 for patients with an inadequate response to 5-aminosalicylates. Its label restricts continuous use to 6 months and it is not indicated for maintenance, reflecting residual theoretical concern about pan-α4 blockade. Carotegrast methyl nonetheless represents proof of principle that integrin blockade can be achieved orally. Ontamalimab, a monoclonal antibody against MAdCAM-1, showed activity in phase 2 but development was discontinued for strategic reasons, and oral α4β7 antagonist peptides remain in early development.

## 6 Interleukin-12/23 and selective interleukin-23p19 antagonists

### 6.1 Ustekinumab

Ustekinumab binds the p40 subunit shared by IL-12 and IL-23, preventing engagement of the IL-12Rβ1 receptor chain and blocking both pathways. Efficacy in CD was established in the UNITI programme, comprising UNITI-1 in patients who had failed TNF antagonists, UNITI-2 in those who had failed conventional therapy, and IM-UNITI for maintenance (Feagan et al., 2016), with approval for CD in 2016. The UNIFI trial subsequently established efficacy in UC: week 8 clinical remission occurred in 15.6% of patients receiving ustekinumab compared with 5.3% receiving placebo, and at week 44 remission was maintained by 43.8% of those receiving 90 mg every 8 weeks versus 24.0% with placebo (Sands et al., 2019b), leading to approval for UC in 2019. Induction is weight-based intravenous dosing followed by fixed subcutaneous maintenance (Deepak and Sandborn, 2017). Long-term extension data extending to 4 years show durable symptomatic remission, sustained in 55.2% of patients randomised to subcutaneous maintenance at week 200 (Afif et al., 2024), and the class safety profile has proved favourable: adverse events of infection, cardiovascular and malignant type do occur, but their incidence in controlled trials and registries has not differed materially from placebo or from background population rates. Biosimilar ustekinumab products have now been approved and have entered the market (U.S. Food and Drug Administration, 2023).

### 6.2 Selective IL-23p19 antagonists

Selective inhibition of the p19 subunit of IL-23 leaves IL-12-driven type 1 immunity intact (Figure 2B), which was hypothesised to improve the therapeutic index. That hypothesis has been borne out: three p19 antagonists have now been approved for both UC and CD, and each has demonstrated efficacy in populations that had already failed TNF antagonists.

Risankizumab was approved for CD in 2022 on the basis of the ADVANCE and MOTIVATE induction trials and the FORTIFY maintenance trial. In ADVANCE, week 12 clinical remission was achieved by 45% of patients receiving risankizumab 600 mg intravenously versus 25% with placebo (adjusted difference 21%, 95% CI 12–29); in the biologic-refractory MOTIVATE population the corresponding figures were 42% and 19% (D'Haens et al., 2022; Ferrante et al., 2022). A fully subcutaneous induction regimen was subsequently evaluated in the phase 3 AFFIRM study, reported in March 2026, in which week 12 clinical remission was achieved by 55% versus 30% with placebo and endoscopic response by 44% versus 14% in a predominantly treatment-refractory population; a supplemental application was filed with the FDA in April 2026 (AbbVie, 2026). Approval for UC followed in June 2024, based on INSPIRE and COMMAND: week 12 clinical remission occurred in 20.3% versus 6.2% with placebo, with endoscopic improvement in 36.5% versus 12.1%, and at week 52 clinical remission was maintained in 40.2% and 37.6% of patients receiving 180 mg and 360 mg subcutaneously respectively, versus 25.1% with placebo (Louis et al., 2024).

Mirikizumab, which had shown dose-dependent activity in a phase 2 study (Sandborn et al., 2020b), became the first p19 antagonist approved for UC, in October 2023, on the basis of LUCENT-1 and LUCENT-2. Week 12 clinical remission occurred in 24.2% of treated patients versus 13.3% with placebo, and week 52 remission in 49.9% versus 25.1%; serious adverse events were less frequent with active treatment than with placebo (3.3% versus 7.8%) (D'Haens et al., 2023). The LUCENT-3 open-label extension reported that, among patients in clinical remission at week 52, 70.1% remained in clinical remission at week 152 by modified non-responder imputation; the 4-year report from the same extension confirmed durable clinical, endoscopic and histological benefit in ulcerative colitis, with generally similar results in biologic-failed and biologic-naive subgroups (Sands et al., 2025; Sands et al., 2026). Approval for CD followed in January 2025 on the basis of VIVID-1, a treat-through trial notable for including ustekinumab as an active reference arm, in which mirikizumab met both co-primary composite endpoints against placebo; the trial was not powered for a formal comparison with ustekinumab (Ferrante et al., 2024). In October 2025 a single-injection, once-monthly subcutaneous maintenance regimen was approved for UC, simplifying administration.

Guselkumab binds p19 and additionally engages CD64 on myeloid cells that produce IL-23, a feature that distinguishes it pharmacologically from the other agents in the class. It was approved for UC in 2024 following the QUASAR induction and maintenance programme (Rubin et al., 2025a) and for CD in March 2025 following GALAXI-2 and GALAXI-3, in which superiority to ustekinumab across endoscopic endpoints was shown in pre-specified pooled analyses of the two trials rather than as the primary comparison within either (Panaccione et al., 2025), and GRAVITI, which validated a fully subcutaneous induction regimen (Hart et al., 2025). In GRAVITI, week 12 clinical remission was achieved by 56.1% of patients receiving guselkumab 400 mg subcutaneously versus 21.4% with placebo, with endoscopic response in 41.3% versus 21.4%. Guselkumab is currently the only IL-23 antagonist offering both intravenous and subcutaneous induction options.

Brazikumab, a further p19 antagonist that had shown promising phase 2 results, was discontinued during phase 3 development for commercial rather than scientific reasons—an illustration of how competitive dynamics in a crowded class now shape the therapeutic landscape as much as trial results do. An unrelated approach to this pathway, the small-molecule PIKfyve inhibitor APY0201, which suppresses IL-12 and IL-23 production upstream, showed activity in murine colitis but has not advanced clinically (Hayakawa et al., 2014).

## 7 Janus kinase inhibitors

### 7.1 Tofacitinib

Tofacitinib, a first-in-class oral inhibitor with activity against JAK1 and JAK3, was the first small molecule of this type approved for UC. In OCTAVE Induction 1, remission at week 8 occurred in 18.5% of patients receiving 10 mg twice daily versus 8.2% with placebo, and in OCTAVE Induction 2 in 16.6% versus 3.6%; in OCTAVE Sustain, week 52 remission occurred in 34.3% and 40.6% of patients receiving 5 mg and 10 mg twice daily respectively, versus 11.1% with placebo (Sandborn et al., 2017). Patient-reported outcome analyses confirmed benefit across symptom domains (Panés et al., 2015). Efficacy is independent of prior TNF antagonist exposure, and the onset of action is rapid—a meaningful advantage in patients with a high symptom burden. The RIVETING study supports dose de-escalation to 5 mg twice daily in patients in stable remission, although month 6 remission was numerically lower after reduction (77.1% versus 90.0%) (Vermeire et al., 2021). Tofacitinib failed to meet its endpoints in CD, a discrepancy that remains incompletely explained.

### 7.2 Upadacitinib

Upadacitinib is a selective JAK1 inhibitor and is the only agent in this class approved for both diseases. Selectivity in this class is relative rather than absolute, and is both assay-dependent and concentration-dependent. Reported fold-selectivity for JAK1 over the other Janus kinases differs substantially between cell-free enzymatic assays and engineered cellular systems, and the margin over JAK2 is consistently narrower than that over JAK3; upadacitinib is JAK1-selective on both measures, whereas tofacitinib has a more balanced JAK1/JAK3 profile. Because fold-selectivity is as much a property of the assay as of the molecule, the functional selectivity achieved in patients depends on the plasma concentration attained; at higher doses the margin over JAK2 narrows, which is one reason why haematological and lipid effects are dose-dependent and why the safety signals discussed below are best considered class effects modified by exposure rather than properties of individual molecules. In UC, the U-ACHIEVE and U-ACCOMPLISH induction trials showed week 8 clinical remission in 26.1% of patients receiving 45 mg once daily versus 4.8% with placebo in U-ACHIEVE, and in U-ACHIEVE Maintenance week 52 remission was achieved by 40.4% and 53.6% of patients receiving 15 mg and 30 mg respectively, versus 10.8% with placebo (Danese et al., 2022b; Vermeire et al., 2023). In CD, U-EXCEL and U-EXCEED showed week 12 clinical remission in 49.5% versus 29.1% and 38.9% versus 21.1% respectively, with endoscopic response in 45.5% versus 13.1% and 34.6% versus 3.5%; in U-ENDURE, week 52 clinical remission was maintained in 37.3% and 47.6% of patients receiving 15 mg and 30 mg versus 15.1% with placebo (Loftus et al., 2023). These are among the largest absolute treatment effects reported for any advanced therapy, and network meta-analyses consistently place upadacitinib in the highest-efficacy tier for UC (Singh et al., 2024). Both programmes were preceded by dose-ranging phase 2 studies in CD and UC respectively (Sandborn et al., 2020c; Sandborn et al., 2020d).

### 7.3 Other Janus kinase and tyrosine kinase 2 inhibitors

Filgotinib, a JAK1-selective agent, showed efficacy in UC in the SELECTION programme and is approved in the European Union, but the United States application was withdrawn (Feagan et al., 2021). It was also evaluated in CD following a positive phase 2 signal in FITZROY (Vermeire et al., 2017), but development in that indication has not been pursued further. Ivarmacitinib, a JAK1 inhibitor developed in China, produced week 8 clinical remission in 22.0%–24.4% of patients versus 4.9% with placebo across three dose groups in the phase 2 AMBER2 trial (Chen et al., 2022). It is a notable example of an advanced therapy developed primarily within an Asian population. Its regulatory position warrants precise statement: the approvals granted by the Chinese National Medical Products Administration in 2025 covered ankylosing spondylitis, rheumatoid arthritis, atopic dermatitis and severe alopecia areata, and the ulcerative colitis programme remained in phase 3 at the time of writing (Keam, 2025). Peficitinib, which is relatively JAK3-selective, produced clinical remission in 27.3% of patients versus 7.0% with placebo, although C-reactive protein was not consistently reduced and a clear dose–response was not established (Sands et al., 2018); it is licensed only for rheumatoid arthritis in Japan and has not been developed further in IBD.

Two strategies have sought to decouple efficacy from systemic exposure. Gut-selective inhibition was pursued with TD-1473 (izencitinib), an oral pan-JAK inhibitor with minimal systemic bioavailability that showed promising phase 1b signals (Sandborn et al., 2020e) but did not separate convincingly from placebo in phase 2b—suggesting that some systemic exposure may be required for efficacy. Deucravacitinib inhibits tyrosine kinase 2 allosterically, binding the regulatory JH2 pseudokinase domain rather than the catalytic site and thereby sparing JAK1/2/3; it showed activity in early UC studies but ultimately missed its primary endpoints in all three phase 2 IBD trials—LATTICE-CD, LATTICE-UC and a supporting study—amid high placebo response rates, and two were terminated early (D'Haens et al., 2025). The rationale for sparing JAK1, JAK2 and JAK3 remains mechanistically attractive, and the compound is approved in psoriasis; whether it can be translated to IBD remains open.

### 7.4 Safety: recalibration after ORAL Surveillance

ORAL Surveillance has had a substantial influence on the positioning of Janus kinase inhibitors in IBD. In this post-authorisation safety trial, conducted in patients with rheumatoid arthritis aged 50 years or older with at least one cardiovascular risk factor, tofacitinib failed to demonstrate non-inferiority to TNF antagonists for major adverse cardiovascular events (3.4% versus 2.5%) and malignancy (4.2% versus 2.9%; hazard ratio for cancers excluding non-melanoma skin cancer 1.48 with 5 mg and 1.47 with 10 mg), with additional signals for venous thromboembolism and all-cause mortality (Ytterberg et al., 2022). The FDA applied a class-wide boxed warning to tofacitinib, baricitinib and upadacitinib covering serious infection, mortality, malignancy, major adverse cardiovascular events and thrombosis, and restricted use to patients with an inadequate response to one or more TNF antagonists; the EMA imposed comparable risk-minimisation measures (U.S. Food and Drug Administration, 2021).

Three qualifications are essential to correct interpretation. First, the trial population was not an IBD population: patients with IBD are on average considerably younger, and the enrichment criteria of ORAL Surveillance selected a high-cardiovascular-risk cohort. Second, IBD-specific analyses have generally found lower absolute event rates: integrated safety analyses of the tofacitinib ulcerative colitis programme, spanning up to 7.8 years of exposure, report low adjudicated incidence of major adverse cardiovascular events, venous thromboembolism and malignancy, with age and pre-existing cardiovascular risk factors emerging as the dominant modifiers (Sandborn et al., 2019; Sandborn et al., 2023a). This evidence is nevertheless dominated by a single molecule in ulcerative colitis, and long-term data for upadacitinib remain shorter. Third, the comparator was an active drug rather than placebo, so the findings describe relative rather than absolute hazard. The pragmatic synthesis now reflected in guidelines is that JAK inhibitors should be used with caution in patients over 65, in current or former smokers, and in those with established cardiovascular disease or a history of thromboembolism or malignancy, but that in younger patients without these features the benefit–risk balance remains favourable—particularly given the magnitude of efficacy demonstrated by upadacitinib. Herpes zoster risk is consistently elevated across the class, and recombinant zoster vaccination before initiation is recommended.

## 8 Sphingosine-1-phosphate receptor modulators

S1P is a bioactive sphingolipid that signals through five G protein-coupled receptors. Functional antagonism of S1P1 causes receptor internalisation and degradation, rendering lymphocytes unable to follow the S1P gradient out of lymph nodes; the resulting sequestration reduces the circulating pool of gut-homing lymphocytes (Wiendl et al., 2021). The class is orally administered, has no immunogenicity, and is reversible on withdrawal.

Fingolimod, the first-generation agent, binds S1P1, S1P3, S1P4 and S1P5 and was limited by bradyarrhythmia attributable to S1P3 engagement; it was not developed for IBD. Ozanimod, selective for S1P1 and S1P5, was approved for UC in 2021 on the basis of the TOUCHSTONE and True North trials: in True North, week 10 clinical remission occurred in 18.4% of treated patients versus 6.0% with placebo, with clinical response in 48% versus 26% and endoscopic improvement in 27% versus 12% (Sandborn et al., 2021). Open-label extension data confirm durability of effect to approximately 3 years, with annual endoscopic and histological assessment—an unusually rigorous long-term dataset. The YELLOWSTONE programme is evaluating ozanimod in CD.

Etrasimod, selective for S1P1, S1P4 and S1P5, was approved for UC in October 2023 following ELEVATE UC 52 and ELEVATE UC 12. Week 12 clinical remission occurred in 27.0% of treated patients versus 7.4% with placebo in ELEVATE UC 52, and remission was sustained at week 52 in 32.1% versus 6.7%; ELEVATE UC 12 showed week 12 remission in 24.8% versus 15.2% (Sandborn et al., 2023c). The molecule was designed for selectivity over S1P_3_, the receptor implicated in the bradyarrhythmia of first-generation agents (Buzard et al., 2014). Etrasimod requires no dose titration, which simplifies initiation relative to ozanimod. The CULTIVATE programme is evaluating etrasimod in CD.

### 8.1 Safety and monitoring

Class-specific monitoring requirements distinguish these agents from biologics and are summarised in Table 4. A baseline electrocardiogram is required because of first-dose bradycardia and atrioventricular conduction delay; ophthalmological assessment is advised in patients with diabetes, uveitis or pre-existing retinal disease because of the risk of macular oedema; liver function should be monitored; varicella zoster serology should be checked and vaccination given if seronegative; and interactions with drugs metabolised through CYP2C8 and CYP2C9, and with agents that slow atrioventricular conduction, must be considered. The requirement for cardiac and ophthalmological oversight represents a meaningful practical burden that should inform patient selection.

## 9 Emerging mechanisms

Three late-stage strategies have generated positive data since 2024, each addressing a different limitation of current therapy: a target not previously addressed pharmacologically in IBD (TL1A), a mechanism that augments endogenous regulation rather than blocking a cytokine (obefazimod), and oral delivery of blockade of an established pathway (icotrokinra) (Table 5). The evidence status of each is indicated in Table 5; findings that rest on conference presentations or company announcements are identified as such throughout this section.

**TABLE 5:** Selected late-stage investigational agents and recently discontinued programmes in inflammatory bowel disease.

Agent	Mechanism	Route	Stage	Principal reported efficacy	Evidence status
Tulisokibart (MK-7240)	Anti-TL1A monoclonal antibody	IV then SC	Phase 3 (UC and CD)	ARTEMIS-UC phase 2: clinical remission at week 12 in 26% vs. 1% with placebo; 32% vs. 11% in a population prospectively enriched by a genetic response biomarker. Phase 3 ATLAS-UC induction-only study reported on 22 June 2026 to have met its primary and key secondary endpoints; numerical results not yet disclosed	Phase 2 peer-reviewed; phase 3 topline by company announcement
Duvakitug (TEV-′574)	Anti-TL1A monoclonal antibody	SC	Phase 3 (UC and CD)	RELIEVE UCCD phase 2b: UC clinical remission at week 14 in 36% and 48% vs. 20% with placebo; CD endoscopic response in 26.1% and 47.8% vs. 13.0%; durable efficacy at 44 weeks	Conference presentation and company disclosure
Obefazimod (ABX464)	Selective enhancer of miR-124 biogenesis; suppresses STAT3, IL-6 and IL-17 signalling	Oral	Phase 3 complete (UC); phase 2b (CD)	ABTECT induction: primary endpoint met in both trials, pooled placebo-adjusted difference 16.4% at week 8. Maintenance: week 44 clinical remission 50.8% (25 mg) and 51.3% (50 mg) vs. 10.4% placebo; 37.2% among induction non-responders continued on 50 mg. Four non-melanoma skin cancers and two other malignancies (both at 50 mg) reported; exposure-adjusted rates within the reported background range for ulcerative colitis	Company disclosure; regulatory submission anticipated
Icotrokinra (JNJ-2113)	Oral peptide antagonist of the IL-23 receptor	Oral	Phase 3 (UC)	ANTHEM-UC phase 2b: clinical response at week 12 in 63.5% vs. 27% with placebo; clinical remission in 30.2% vs. 11.1%, improving through week 28	Company disclosure
Deucravacitinib	Allosteric tyrosine kinase 2 inhibitor	Oral	Phase 2 complete; IBD programme not advanced	Missed the primary endpoint in all three phase 2 IBD studies (LATTICE-CD, LATTICE-UC and a supporting Crohn’s disease study) amid high placebo response; two were terminated early. Allosteric TYK2 inhibition remains mechanistically attractive but is not supported by the IBD data	Peer-reviewed

Data derived from conference presentations and company announcements have not completed peer review and should be interpreted with corresponding caution; definitive figures may change on publication. Entries are current to 18 July 2026. CD, Crohn’s disease; IL, interleukin; IV, intravenous; JAK, Janus kinase; SC, subcutaneous; STAT, signal transducer and activator of transcription; TL1A, TNF-like ligand 1A; TYK2, tyrosine kinase 2; UC, ulcerative colitis.

### 9.1 Anti-TL1A antibodies

TL1A, encoded by *TNFSF15,* signals through death receptor 3 and amplifies effector T cell responses while simultaneously activating intestinal fibroblasts. *TNFSF15* polymorphisms are associated with IBD susceptibility, and—uniquely among current targets—with response to TL1A blockade, which has enabled a genetically guided development programme.

Tulisokibart (MK-7240, formerly PRA023) was evaluated in the phase 2 ARTEMIS-UC trial. Week 12 clinical remission occurred in 26% of treated patients versus 1% with placebo in the first cohort; in a second cohort prospectively enriched using a companion diagnostic for a genetic response signature, remission occurred in 32% versus 11% (Sands et al., 2024). This is the first prospective demonstration in IBD that a genetic biomarker can be used to enrich a trial population. Its predictive clinical utility nevertheless remains to be established: the companion diagnostic has not yet been evaluated prospectively for treatment selection in routine practice, and larger studies will be required before this approach can be regarded as a validated precision-medicine strategy. On 22 June 2026 the phase 3 ATLAS-UC induction-only study was reported to have met its primary endpoint of clinical remission at week 12 together with key secondary endpoints of endoscopic improvement, clinical response and histologic-endoscopic mucosal improvement—the first positive phase 3 induction result for any anti-TL1A antibody in UC. Numerical results were not disclosed in the topline announcement and await presentation; the companion induction-plus-maintenance study and the Crohn’s disease programme (ARES-CD) are ongoing (Merck & Co, 2026).

Duvakitug (TEV‐'574) reported phase 2b results in the RELIEVE UCCD programme in which week 14 clinical remission in UC was achieved by 36% and 48% of patients at two dose levels versus 20% with placebo, with endoscopic response in CD of 26.1% and 47.8% versus 13.0%; 44-week maintenance data reported in February 2026 indicated durable efficacy (Sanofi and Teva Pharmaceutical Industries, 2026). Phase 3 development is proceeding. Its dual anti-inflammatory and anti-fibrotic activity makes it the most plausible current candidate for addressing stricturing CD, for which no medical therapy currently exists.

### 9.2 Obefazimod

Obefazimod (ABX464), evaluated in ulcerative colitis, is a first-in-class oral small molecule that binds the Cap-binding complex and selectively enhances the biogenesis of miR-124, a microRNA that suppresses STAT3, IL-6 and IL-17 signalling. Rather than blocking a single cytokine, it augments an endogenous anti-inflammatory pathway. In the phase 3 ABTECT induction programme, once-daily obefazimod 50 mg met the primary endpoint of week 8 clinical remission in both trials, with a pooled placebo-adjusted difference of 16.4%. Maintenance results announced in June 2026 reported week 44 clinical remission in 50.8% and 51.3% of patients receiving 25 mg and 50 mg respectively, versus 10.4% with placebo (placebo-adjusted differences 39.3% and 40.3%; both p < 0.0001) (Abivax, 2026a). A supplementary analysis of induction non-responders continued on 50 mg reported week 44 clinical remission of 37.2% and endoscopic remission of 34.5% (Abivax, 2026b). These figures require balanced interpretation. A small number of malignancies was reported during maintenance, with exposure-adjusted rates stated by the sponsor to fall within the reported background range for ulcerative colitis populations, and all cases occurred in patients with recognised risk factors, but longer follow-up is needed before this dose can be considered free of a malignancy signal (Abivax, 2026b). A phase 2b programme in CD is ongoing, and a regulatory submission for UC is anticipated. These figures await confirmation at peer review.

### 9.3 Oral interleukin-23 receptor antagonist peptides

Icotrokinra (JNJ-2113) is an orally administered peptide that binds the IL-23 receptor with picomolar affinity, thereby achieving IL-23 pathway blockade without parenteral administration—a combination that has not previously been possible. In the phase 2b ANTHEM-UC study, week 12 clinical response was achieved by 63.5% of patients at the highest dose versus 27% with placebo, and clinical remission by 30.2% versus 11.1%, with responses continuing to improve through week 28 (Johnson & Johnson, 2025). Phase 3 evaluation is under way.

### 9.4 Other approaches and discontinued programmes

Several other strategies merit brief mention. Antisense inhibition of SMAD7 with mongersen produced a positive phase 2 result (Monteleone et al., 2015) that was not replicated in phase 3 (Sands et al., 2020), and the CCR9 antagonist vercirnon did not meet its phase 3 endpoints (Feagan et al., 2015). Allogeneic expanded adipose-derived mesenchymal stem cells (darvadstrocel) were licensed for complex perianal fistulising CD on the strength of ADMIRE-CD (Panés et al., 2016), but the confirmatory ADMIRE-CD II trial did not meet its endpoint and the product was withdrawn from the European market in December 2024 (Buskens and Bemelman, 2026). Faecal microbiota transplantation has shown reproducible but heterogeneous benefit in UC and remains investigational, as do engineered cell therapies. Combination advanced therapy is discussed in Section 10.4.

## 10 Comparative effectiveness and treatment positioning

### 10.1 Head-to-head trials

Until recently, positioning relied entirely on indirect comparison across trials with different populations, endpoints and placebo rates. Randomised trials incorporating active comparators have now begun to provide direct evidence, but their designs differ in a way that materially affects interpretation. In formal head-to-head trials the comparison between the two active agents was itself a pre-specified endpoint powered for superiority or non-inferiority; in trials with an active reference arm the primary comparison was against placebo, and the reference agent was not included with the statistical power required for a formal between-drug comparison (Table 6).

**TABLE 6:** Randomised head-to-head trials of advanced therapies in inflammatory bowel disease.

Trial	Design	Population	Comparison	Primary endpoint	Result
VARSITY	Head-to-head (active comparison powered for superiority)	Moderate-to-severe UC (n = 769); ≤25% prior anti-TNF exposure	Vedolizumab IV vs. adalimumab SC	Clinical remission, week 52	31.3% vs. 22.5% (difference 8.8 pp, 95% CI 2.5–15.0; P = 0.006); endoscopic improvement 39.7% vs. 27.7%; corticosteroid-free remission favoured adalimumab (21.8% vs. 12.6%)
SEAVUE	Head-to-head (active comparison as primary endpoint)	Biologic-naive moderate-to-severe CD (n = 386)	Ustekinumab vs. adalimumab	CDAI clinical remission, week 52	65% vs. 61% (difference 4 pp, 95% CI −6 to 14; P = 0.42); comparable safety
SEQUENCE	Head-to-head (co-primary non-inferiority and superiority)	Moderate-to-severe CD after anti-TNF failure (n = 520)	Risankizumab vs. ustekinumab	Clinical remission (week 24, non-inferiority) and endoscopic remission (week 48, superiority)	Clinical remission 58.6% vs. 39.5% (adjusted difference 18.4 pp, 95% CI 6.6–30.3); endoscopic remission 31.8% vs. 16.2% (adjusted difference 15.6 pp, 95% CI 8.4–22.9; P < 0.001)
GALAXI-2 and GALAXI-3	Active reference (primary comparison vs. placebo; guselkumab vs. ustekinumab shown in pre-specified pooled analyses)	Moderate-to-severe CD, biologic-naive and biologic-exposed	Guselkumab vs. ustekinumab vs. placebo	Composite clinical and endoscopic endpoints	Guselkumab superior to ustekinumab across all pooled endoscopic endpoints
VIVID-1	Active reference (primary comparison vs. placebo; not powered for mirikizumab vs. ustekinumab)	Moderate-to-severe CD, treat-through design	Mirikizumab vs. ustekinumab (active reference) vs. placebo	Composite clinical remission and endoscopic response	Mirikizumab superior to placebo across clinical, endoscopic and patient-reported endpoints
VEGA	Combination proof-of-concept (phase 2; not a between-agent head-to-head)	Moderate-to-severe UC, biologic-naive (phase 2 proof of concept)	Guselkumab plus golimumab vs. either agent alone	Clinical response, week 12	Combination numerically superior for clinical and endoscopic outcomes without an evident excess of adverse events

CDAI, Crohn’s Disease Activity Index; CD, Crohn’s disease; CI, confidence interval; IV, intravenous; pp, percentage points; SC, subcutaneous; TNF, tumour necrosis factor; UC, ulcerative colitis.

VARSITY compared vedolizumab with adalimumab in 769 patients with UC and found vedolizumab superior for week 52 clinical remission (31.3% versus 22.5%; difference 8.8 percentage points, 95% CI 2.5–15.0) and for endoscopic improvement (39.7% versus 27.7%) (Sands et al., 2019a). An important nuance is that corticosteroid-free remission favoured adalimumab (21.8% versus 12.6%), reflecting differences in trial design regarding steroid tapering rather than a true reversal of effect.

SEAVUE compared ustekinumab with adalimumab in biologic-naive CD and found no significant difference in week 52 clinical remission (65% versus 61%; difference 4%, 95% CI −6 to 14), with comparable safety (Sands et al., 2022). The practical implication is that in biologic-naive CD, factors other than efficacy—route of administration, monitoring burden, cost and comorbidity—may reasonably determine choice.

SEQUENCE compared risankizumab with ustekinumab in 520 patients with CD who had failed TNF antagonists. Risankizumab was non-inferior for week 24 clinical remission (58.6% versus 39.5%; adjusted difference 18.4 percentage points, 95% CI 6.6–30.3) and superior for week 48 endoscopic remission (31.8% versus 16.2%; adjusted difference 15.6 percentage points, 95% CI 8.4–22.9) (Peyrin-Biroulet et al., 2024). This is the most directly actionable head-to-head result currently available and supports preferential use of a p19-selective antagonist over ustekinumab after TNF antagonist failure.

GALAXI-2 and GALAXI-3 were placebo-controlled trials incorporating ustekinumab as an active reference; superiority of guselkumab over ustekinumab across endoscopic endpoints was demonstrated in pre-specified pooled analyses of the two trials rather than as the primary comparison within either (Panaccione et al., 2025). VIVID-1 likewise incorporated ustekinumab as an active reference for mirikizumab, and was not powered for a formal comparison between the two active agents (Ferrante et al., 2024). Taken together, these trials indicate that selective p19 blockade is at least as effective as p40 blockade, and that individual p19 antagonists have shown superiority to ustekinumab for specific endoscopic endpoints in the populations studied—risankizumab in patients with prior TNF-antagonist failure, and guselkumab in a mixed population. These findings should not be generalised to a class-wide endoscopic advantage across all clinical settings.

### 10.2 Network meta-analysis and living guidelines

The AGA has adopted a living guideline model for moderate-to-severe UC, underpinned by a continuously updated network meta-analysis that groups advanced therapies into efficacy tiers rather than ranking them individually—an approach that appropriately reflects the imprecision of indirect comparison (Singh et al., 2024). Critically, the guideline groups agents separately according to prior exposure to advanced therapy. For patients naive to advanced therapy it suggests a higher-efficacy medication (infliximab, vedolizumab, ozanimod, etrasimod, upadacitinib, risankizumab or guselkumab) or an intermediate-efficacy medication (golimumab, ustekinumab, tofacitinib, filgotinib or mirikizumab) in preference to a lower-efficacy medication (adalimumab). For patients previously exposed to at least one advanced therapy, particularly a TNF antagonist, it suggests a higher-efficacy medication (tofacitinib, upadacitinib or ustekinumab) or an intermediate-efficacy medication (filgotinib, mirikizumab, risankizumab or guselkumab) in preference to a lower-efficacy medication (adalimumab, vedolizumab, ozanimod or etrasimod). Both are conditional recommendations based on low certainty of evidence, and the groupings differ appreciably between the two populations: only upadacitinib appears in the higher-efficacy group in both, whereas risankizumab moves from the higher to the intermediate group once a patient has been exposed to advanced therapy. The guideline also recommends early use of advanced therapy in preference to gradual step-up care, and recommends against continuing to cycle within a mechanistic class after failure. A parallel living guideline addresses CD. Because these documents are revised as evidence accrues, authors and clinicians should cite the review date rather than the original publication date.

### 10.3 Early advanced therapy and treat-to-target

Two conceptual shifts now frame positioning. The first is treat-to-target. STRIDE-II defines a hierarchy: symptomatic response as an immediate target, normalisation of C-reactive protein and faecal calprotectin as intermediate targets, and symptomatic remission with endoscopic healing and restored quality of life as long-term targets (Turner et al., 2021). Symptom control alone is no longer adequate, because persistent endoscopic inflammation predicts relapse, hospitalisation and surgery.

The second is early advanced therapy. The PROFILE trial randomised patients with newly diagnosed CD to top-down infliximab plus an immunomodulator or to accelerated step-up care. Sustained steroid-free and surgery-free remission was achieved by 79% in the top-down arm versus 15% in the step-up arm, with endoscopic remission in 67% versus 44% (Noor et al., 2024). Notably, the biomarker used to stratify randomisation did not predict differential benefit: top-down therapy was superior across all subgroups. PROFILE provides strong support for early combined immunosuppression in patients with newly diagnosed CD, the population in which it was conducted. It does not establish early advanced therapy as a default for all patients with moderate-to-severe IBD, and its findings have not been replicated in UC or in patients with longstanding disease. Figure 4 integrates these principles into a practical framework.

**FIGURE 4:**
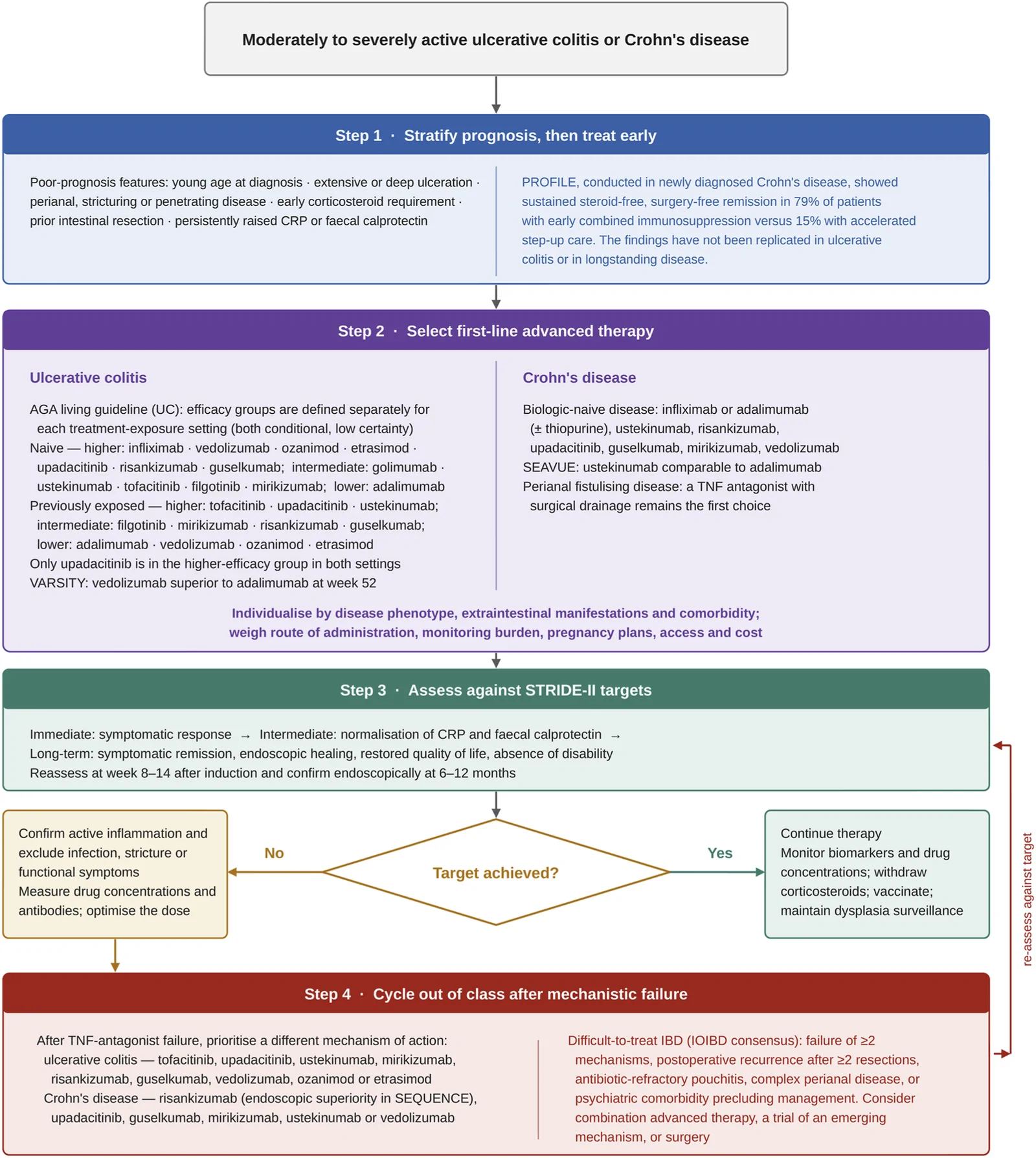
A contemporary treat-to-target framework for positioning advanced therapies in moderately to severely active inflammatory bowel disease, integrating prognostic stratification, early advanced therapy, STRIDE-II targets and mechanism-based cycling after treatment failure.

### 10.4 Combination advanced therapy

If a single mechanism cannot exceed a remission ceiling of roughly 50% in placebo-controlled trials, combining mechanisms is the obvious next step. The VEGA study evaluated induction with guselkumab plus golimumab followed by guselkumab monotherapy in UC and reported numerically higher clinical and endoscopic outcomes than either agent alone, without an evident excess of adverse events (Feagan et al., 2023). The DUET-UC and DUET-CD phase 3 programmes are now testing this combination formally. A systematic review of 30 observational studies comprising 279 patients reported pooled clinical remission of 59% and endoscopic remission of 34% with dual advanced therapy in refractory disease (Ahmed et al., 2022), but these uncontrolled data are subject to substantial selection bias. Until randomised data mature, combination therapy should be reserved for patients with difficult-to-treat disease, ideally within a trial.

### 10.5 Acute severe ulcerative colitis

Acute severe ulcerative colitis is the setting in which treatment decisions are most consequential and the evidence for advanced therapy is thinnest. Roughly one in four patients with UC will experience at least one severe flare, and admission carries a defined risk of colectomy during that hospitalisation (Feuerstein et al., 2020). Management therefore differs in kind, not merely in degree, from the elective positioning discussed above: decisions are made over days rather than months, the endpoint that matters is avoidance of colectomy rather than endoscopic remission, and almost none of the pivotal trials summarised in Tables 2 and 3 enrolled these patients.

Assessment begins with confirmation of severity and exclusion of superimposed infection. Stool testing for *Clostridioides difficile* and flexible sigmoidoscopy with biopsy for cytomegalovirus should precede escalation, because both alter management and both are more common in this population than in ambulatory disease (Raine et al., 2022). Abdominal radiography identifies colonic dilatation. Intravenous corticosteroids remain first-line, but their limits are well defined: a systematic review and meta-regression of some 2,000 patients found an overall response rate of 67%, of whom 27% nonetheless required colectomy in the short term, and neither figure improved across three decades of published series (Turner et al., 2007).

Because most steroid failures declare themselves early, formal assessment on day 3 is the pivot of management. The Oxford index identifies patients passing more than eight stools daily, or three to eight stools with a C-reactive protein above 45 mg/L, as being at approximately 85% risk of colectomy during that admission (Travis et al., 1996). The Edinburgh (Ho) score incorporates stool frequency, colonic dilatation and an albumin below 30 g/L, and performs comparably (Ho et al., 2004). Neither index is a decision rule in itself, but both identify the patients in whom rescue therapy should begin immediately rather than after a further period of observation, and delay at this point is associated with worse surgical outcomes.

Two rescue options have randomised support. Ciclosporin was established in a small placebo-controlled trial in which response occurred in 82% of treated patients versus none receiving placebo (Lichtiger et al., 1994). Infliximab entered the same space subsequently, and the two have been compared directly: CYSIF found no significant difference in treatment failure at day 98, and the pragmatic CONSTRUCT trial found no difference in quality-adjusted survival, colectomy rates, serious adverse events or cost-effectiveness (Laharie et al., 2012; Williams et al., 2016). Choice is therefore governed by local experience, prior thiopurine exposure—ciclosporin is generally reserved for thiopurine-naive patients because it is a bridge to thiopurine maintenance—and the intended long-term strategy. Sequential rescue, in which one agent follows failure of the other, carries a high complication rate and should be undertaken only in centres with immediate surgical support.

Accelerated infliximab dosing is widely used on the rationale set out in Section 4.3: severe colitis increases faecal drug loss and lowers exposure at precisely the point when the inflammatory burden is greatest, and observational series report lower early colectomy rates with intensified regimens (Gibson et al., 2015). The randomised PREDICT-UC trial, however, did not demonstrate superiority of intensified induction over standard dosing (Choy et al., 2024), so the practice remains reasonable but unproven, and dose intensification should not substitute for timely surgical assessment.

The role of JAK inhibitors is expanding rapidly but rests entirely on observational data. A matched case-control study found that tofacitinib reduced the 90-day colectomy risk in biologic-experienced hospitalised patients, with the signal strongest at the highest dose intensity (Berinstein et al., 2021) and multicentre experience with upadacitinib has reported 90-day colectomy rates of approximately one in four in a comparably refractory population (Berinstein et al., 2024). Systematic reviews consistently emphasise that no randomised trial of a JAK inhibitor in this setting has been completed (Gisbert and Chaparro, 2025). Rapid onset is advantageous when a decision must be made within days, but three cautions apply: venous thromboembolic risk deserves particular attention in patients who are immobile, dehydrated and severely inflamed, even though event rates in the tofacitinib UC programme were low and largely confined to those with additional risk factors (Sandborn et al., 2019); pharmacological thromboprophylaxis is indicated in all patients admitted with acute severe colitis despite rectal bleeding; and the absence of randomised evidence should be disclosed when these agents are used outside a trial.

Throughout, early surgical consultation is part of good care rather than an admission of medical failure. Colectomy performed electively after failed rescue has substantially better outcomes than colectomy performed as an emergency after prolonged medical temporising, and current society guidance emphasises joint medical and surgical decision-making from the day of admission (Rubin et al., 2019; Raine et al., 2022).

## 11 Unmet needs and future directions

### 11.1 The therapeutic ceiling

Figure 3 shows greater similarity between agents than difference. Despite mechanisms as distinct as cytokine neutralisation, trafficking blockade, kinase inhibition and lymphocyte sequestration, induction remission rates cluster between roughly 15% and 50%, placebo-adjusted differences rarely exceed 25 percentage points, and endoscopic remission rates are consistently lower still (Table 7). Several explanations are plausible and not mutually exclusive: trial populations are enriched for refractory disease; the placebo response is substantial; established fibrosis and epithelial damage are not reversible by immunosuppression; and IBD is almost certainly not one disease but several, with distinct dominant pathways masked by clinical classification. A further consideration is methodological: the rates shown derive from trials that differ substantially in population, endpoint definition and design, and treat-through studies are not directly comparable with responder-enriched randomised-withdrawal maintenance studies. The apparent ceiling may therefore reflect trial design and patient selection in part, rather than a clearly established biological limit of current therapy. Breaking it will probably require patient stratification rather than more potent immunosuppression.

**TABLE 7:** Clinical and endoscopic outcomes reported together in pivotal trials of advanced therapy.

Trial (agent)	Endoscopic endpoint and definition	Week	Clinical remission active vs. comparator	Endoscopic outcome active vs. comparator	Design
Ulcerative colitis					
ACT 1 (infliximab 5 mg/kg)	Mucosal healing; MES 0 or 1	8	38.8% vs. 14.9%	62.0% vs. 33.9%	Treat-through
ULTRA 2 (adalimumab)	Mucosal healing; MES ≤1	8/52	16.5% vs. 9.3%	41.1% vs. 31.7% (week 8); 25.0% vs. 15.4% (week 52)	Treat-through
GEMINI 1 (vedolizumab)	Mucosal healing; MES 0 or 1	6/52	16.9% vs. 5.4%	40.9% vs. 24.8% (week 6); 51.6% (q8w), 56.0% (q4w) vs. 19.8% (wk 52)	Induction; randomised withdrawal
VISIBLE 1 (vedolizumab SC)	Mucosal healing; MES ≤1	52	46% vs. 14%	56.6% vs. 21.4%	Randomised withdrawal
UNIFI (ustekinumab)	Endoscopic improvement; MES 0 or 1	8/44	15.6% vs. 5.3%; 43.8% vs. 24.0%	26.3%, 27.0% vs. 13.8% (week 8); 43.6% (q12w), 51.1% (q8w) vs. 28.6% (week 44)	Induction; randomised withdrawal
OCTAVE Induction 1 (tofacitinib 10 mg BID)	Mucosal healing; MES ≤1	8	18.5% vs. 8.2%	31.3% vs. 15.6%	Induction
OCTAVE Induction 2 (tofacitinib 10 mg BID)	Mucosal healing; MES ≤1	8	16.6% vs. 3.6%	28.4% vs. 11.6%	Induction
OCTAVE Sustain (tofacitinib)	Mucosal healing; MES ≤1	52	34.3% (5 mg), 40.6% (10 mg) vs. 11.1%	37.4% (5 mg), 45.7% (10 mg) vs. 13.1%	Randomised withdrawal
U-ACHIEVE/U-ACCOMPLISH (upadacitinib 45 mg)	Endoscopic improvement; MES ≤1	8	26.1% vs. 4.8%	36% (U-ACHIEVE), 44% (U-ACCOMPLISH) vs. 7%–8%	Induction
True North (ozanimod)	Endoscopic improvement; MES ≤1	10/52	18.4% vs. 6.0%	27% vs. 12% (week 10); 46% vs. 26% (week 52)	Induction; randomised withdrawal
ELEVATE UC 52 (etrasimod)	Endoscopic improvement; MES ≤1	12/52	27.0% vs. 7.4%; 32.1% vs. 6.7%	35% (96/274) vs. 14% (19/135) at week 12; 37% (102/274) vs. 10% (14/135) at week 52	Treat-through
ELEVATE UC 12 (etrasimod)	Endoscopic improvement; MES ≤1	12	24.8% vs. 15.2%	31% (68/222) vs. 19% (21/112)	Induction
LUCENT-1 (mirikizumab)	Endoscopic remission; MES 0 or 1	12	24.2% vs. 13.3%	36.3% vs. 21.1%	Induction
LUCENT-2 (mirikizumab 200 mg SC)	Endoscopic remission; MES 0 or 1 (excluding friability)	40 (52 total)	49.9% vs. 25.1%	58.6% vs. 29.1%	Randomised withdrawal
INSPIRE (risankizumab 1,200 mg IV)	Endoscopic improvement; MES ≤1	12	20.3% vs. 6.2%	36.5% vs. 12.1%	Induction
COMMAND (risankizumab SC)	Endoscopic improvement; MES ≤1	52	40.2% (180 mg), 37.6% (360 mg) vs. 25.1%	50.8% (180 mg), 48.3% (360 mg) vs. 31.7%	Randomised withdrawal
QUASAR (guselkumab)	Endoscopic improvement; MES ≤1 Endoscopic normalisation; MES 0	12/44	See text	Improvement 37% vs. 13% (week 12); normalisation 15% vs. 5% (week 12); Normalisation 34% (200 q4w), 35% (100 q8w) vs. 15% (week 44)	Induction; randomised withdrawal
VARSITY (vedolizumab vs. adalimumab)	Endoscopic improvement; MES ≤1	52	31.3% vs. 22.5%	39.7% vs. 27.7%	Head-to-head
LIBERTY-UC (infliximab SC)	No standalone endoscopic endpoint. Composite endoscopic–histologic mucosal improvement (MES 0–1 plus RHI ≤3)	54	43.2% vs. 20.8%	Composite 35.7% vs. 16.7%	Randomised withdrawal
Crohn’s disease					
ADVANCE (risankizumab 600 mg IV)	Endoscopic response; SES-CD decrease >50%	12	45% vs. 25%	40% vs. 12%	Induction
MOTIVATE (risankizumab 600 mg IV)	Endoscopic response; SES-CD decrease >50%	12	42% vs. 19%	29% vs. 11%	Induction
FORTIFY (risankizumab SC)	Endoscopic response; SES-CD decrease >50% (co-primary endpoint)	52	See text	47% (180 mg), 46% (360 mg) vs. 21%	Randomised withdrawal
U-EXCEL (upadacitinib 45 mg)	Endoscopic response; SES-CD decrease >50% (or ≥2 points if baseline 4)	12	49.5% vs. 29.1%	45.5% vs. 13.1%	Induction
U-EXCEED (upadacitinib 45 mg)	Endoscopic response; SES-CD decrease >50%	12	38.9% vs. 21.1%	34.6% vs. 3.5%	Induction
U-ENDURE (upadacitinib)	Endoscopic response; SES-CD decrease >50%	52	37.3% (15 mg), 47.6% (30 mg) vs. 15.1%	27.6% (15 mg), 40.1% (30 mg) vs. 7.3%	Randomised withdrawal
GRAVITI (guselkumab 400 mg SC)	Endoscopic response; SES-CD decrease ≥50% (co-primary endpoint)	12	56.1% vs. 21.4%	41.3% vs. 21.4%	Treat-through
GALAXI-2 and GALAXI-3 (guselkumab)	Endoscopic response; SES-CD decrease ≥50% Endoscopic remission; SES-CD ≤4, ≥2-point reduction, no subscore > 1	48	See text	Pooled analysis vs. ustekinumab: response 52.7% (200 q4w), 47.9% (100 q8w) vs. 37.1%; Remission 37.2%, 33.2% vs. 24.7%	Treat-through; ustekinumab active reference
VIVID-1 (mirikizumab)	Endoscopic response; SES-CD decrease ≥50%	52	See text	38% vs. 9% (placebo)	Treat-through; ustekinumab active reference
SEQUENCE (risankizumab vs. ustekinumab)	Endoscopic remission; SES-CD ≤4, ≥2-point reduction, no subscore > 1	48	58.6% vs. 39.5% (week 24)	31.8% vs. 16.2%	Head-to-head
SEAVUE (ustekinumab vs. adalimumab)	Endoscopic remission (SES-CD-based)	52	65% vs. 61%	29% vs. 31% (not significant)	Head-to-head
LIBERTY-CD (infliximab SC)	Endoscopic response; SES-CD decrease ≥50% (co-primary endpoint)	54	62.3% vs. 32.1%	51.1% vs. 17.9%	Randomised withdrawal
AFFIRM (risankizumab SC induction)	Endoscopic response; SES-CD decrease ≥50% (co-primary endpoint; company announcement, not yet peer-reviewed)	12	55% vs. 30%	44% vs. 14%	Induction

Endoscopic endpoint definitions differ systematically between indications and between trials: ulcerative colitis trials use the Mayo endoscopic subscore (MES) and Crohn’s disease trials the Simple Endoscopic Score for Crohn’s Disease (SES-CD), and the threshold applied to each varies. Denominators also differ: in treat-through designs all randomised patients contribute, whereas in randomised-withdrawal maintenance designs only induction responders are re-randomised, so maintenance rates from the two designs are not directly comparable. The table is therefore intended to display the consistent gap between symptomatic and endoscopic outcomes rather than to support comparison between trials. Two trials that might otherwise be expected here are omitted or qualified: VISIBLE 2 (subcutaneous vedolizumab in Crohn’s disease) reported no endoscopic endpoint, all rank-ordered outcomes being based on the Crohn’s Disease Activity Index; and LIBERTY‐UC reported no standalone endoscopic endpoint, its endoscopic data forming part of a composite endoscopic–histologic endpoint. For ELEVATE UC 52 and ELEVATE UC 12 the composite endpoint of endoscopic improvement with histologic remission reported in those trials is a separate outcome and is not shown here. For GALAXI‐2 and GALAXI‐3, the guselkumab versus ustekinumab comparison was prespecified in the pooled dataset rather than within either trial individually. IV, intravenous; MES, Mayo endoscopic subscore; RHI, Robarts Histopathology Index; SC, subcutaneous; SES-CD, Simple Endoscopic Score for Crohn’s Disease.

### 11.2 Precision medicine

Three biomarker strategies are at different stages of translation. Pharmacogenomic prediction of immunogenicity through HLA-DQA1*05 genotyping is the most immediately implementable, with a clear action—combination therapy or an alternative mechanism in carriers (Sazonovs et al., 2020). Prospective genetic enrichment for response, demonstrated for the first time in ARTEMIS-UC (Sands et al., 2024), is the most conceptually significant, because it inverts the usual sequence in which biomarkers are sought *post hoc*. Transcriptomic signatures such as mucosal oncostatin M predict TNF antagonist non-response retrospectively—in the derivation cohorts, complete mucosal healing on infliximab was achieved by about 85% of patients with low oncostatin M but only 10%–15% of those with high expression—but await prospective validation (West et al., 2017); the PROFILE experience, in which a promising T cell signature failed to predict differential benefit, illustrates how frequently such markers fail translation. A fourth line of investigation concerns the gut microbiome, whose relationship with advanced therapy appears to be bidirectional. Baseline microbial features, including community richness and the abundance of secondary bile acid-producing taxa, have been associated with subsequent response to anti-cytokine therapy, vedolizumab and ustekinumab, while effective therapy in turn shifts the microbiota towards profiles resembling those of healthy controls (Becker et al., 2023; Lazarević et al., 2025). These associations should be interpreted cautiously: the cohorts are small, independent replication is scarce, and disease activity, diet and concomitant medication are difficult to disentangle from treatment effect. A prospective study of 79 patients with an external replication cohort of 47 found that previously reported microbial predictors did not replicate and added little to clinical variables alone (Prins et al., 2024), so microbiome profiling remains mechanistically informative rather than clinically actionable. A related question is whether the epithelial barrier can be targeted directly. Larazotide acetate, an oral zonulin antagonist that restores tight-junction integrity, reached phase 3 in coeliac disease before development was discontinued for futility in 2022, and it has not entered a registered clinical trial in IBD; no barrier-directed agent has yet reached advanced clinical development in this indication, and barrier restoration remains an area of preclinical interest without clinical proof of concept.

### 11.3 Special populations and unresolved questions

Several groups remain poorly served. Older patients face higher infection and malignancy risks and are systematically under-represented in trials; gut-selective agents such as vedolizumab and the IL-23 class are generally preferred, and JAK inhibitors used with caution. Stricturing CD has no licensed medical therapy, which is why the anti-fibrotic potential of TL1A blockade is of particular interest. Perianal fistulising CD remains dependent on TNF antagonists combined with surgical drainage, and the withdrawal of darvadstrocel has removed the only licensed cell therapy (Buskens and Bemelman, 2026). Evidence in pregnancy is strongest for TNF antagonists and vedolizumab; data for the newer oral agents are limited, and the small molecules are generally avoided. Paediatric labelling lags adult approval by several years for most agents.

Finally, an international consensus has formalised the concept of difficult-to-treat IBD, defined by failure of advanced therapies with at least two different mechanisms of action, or by postoperative recurrence after two or more resections, antibiotic-refractory pouchitis, complex perianal disease, or psychiatric comorbidity precluding adequate management (Parigi et al., 2023). This is a growing population for which no evidence-based sequence exists.

### 11.4 Advanced therapy in newly industrialised nations

Almost all of the evidence summarised in this review was generated in North America, Europe and, to a lesser extent, Japan. Yet incidence is now rising fastest in newly industrialised nations, and China alone is projected to move from accelerating incidence into compounding prevalence around 2030 (Shao et al., 2022). The populations in which the advanced-therapy armamentarium is expanding most rapidly are therefore not the populations in which it was developed, and three considerations modify positioning in these settings in ways that guidelines written for regions where tuberculosis and hepatitis B are not endemic, and where resources are less constrained, do not address.

The first is endemic infection, and it is the most clinically consequential. Tuberculosis and hepatitis B remain prevalent across much of Asia, and the choice of mechanism therefore carries implications that do not arise in settings where these infections are not endemic. TNF antagonists confer a substantially higher risk of reactivating latent tuberculosis than ustekinumab or vedolizumab, and Asian consensus statements accordingly require an interferon-γ release assay or tuberculin skin testing together with chest radiography before initiation, with repeat testing during treatment in high-burden regions (Park et al., 2018). Hepatitis B surface antigen and core antibody should be tested in all patients, with antiviral prophylaxis for surface-antigen carriers continued for at least 6 months after the last dose of immunosuppression; occult infection identified by isolated core antibody positivity requires monitoring rather than routine prophylaxis (Ooi et al., 2019). The practical consequence is that where screening capacity, interferon-γ release assay availability or antiviral access is constrained, a gut-selective or IL-23-directed agent may be preferable to a TNF antagonist on infection-risk grounds alone—a positioning logic essentially absent from North American and European guidance, in which the TNF antagonists’ cost advantage usually dominates.

The second is that this divergence arises from context rather than from biology. East Asian subgroup analyses of the pivotal programmes have generally shown efficacy consistent with the global populations: subgroup analyses of the tofacitinib phase 3 programme in East Asian patients reported induction and maintenance effects comparable to the overall trial populations (Motoya et al., 2018), and a similar concordance has been reported for ustekinumab in the UNIFI East Asian subpopulation (Hisamatsu et al., 2021). A dedicated Japanese registration trial of vedolizumab in ulcerative colitis reproduced the direction of effect seen globally (Motoya et al., 2019); the corresponding Crohn’s disease trial did not meet its primary endpoint in a smaller sample, and its comparative analyses were exploratory (Watanabe et al., 2020). There is therefore no evidence base for assuming attenuated efficacy in Asian populations, and dose or expectation adjustments on ethnic grounds are not warranted.

The third is access, which in practice has been the binding constraint more often than evidence. In China, the inclusion of adalimumab in the National Reimbursement Drug List in 2019 and infliximab in 2020 was followed by steep price reductions and a marked rise in uptake; biosimilar entry has compounded the effect, with multiple infliximab and adalimumab biosimilars now approved (Du et al., 2024). Before listing, high out-of-pocket costs limited both initiation and treatment duration, and premature discontinuation for financial reasons remains a recognised cause of loss of response that has no counterpart in the trial literature. Newer mechanisms typically reach reimbursement several years after regulatory approval, so the practical sequence available to a clinician in Chengdu or Jakarta may differ from the guideline sequence not because of any disagreement about the evidence, but because half of the recommended options are unaffordable.

These three considerations do not call for separate guidelines so much as for explicit acknowledgement that positioning is context-dependent. A framework that begins with efficacy tiers and adds safety, route and cost as secondary modifiers describes practice in high-resource settings reasonably well. In high-burden, resource-constrained settings the ordering is closer to the reverse: infection risk and reimbursement status frequently determine the shortlist, and efficacy discriminates within it. Reviews and guidelines that do not state this leave the majority of the world’s incident patients without applicable guidance.

### 11.5 Access, cost and equity

The expansion of therapeutic choice has been accompanied by cost escalation that limits access in most of the world. Biosimilar infliximab and adalimumab have reduced acquisition costs substantially (Jørgensen et al., 2017), and ustekinumab biosimilars are now following (U.S. Food and Drug Administration, 2023). The tension between the guideline recommendation to use a higher-efficacy agent first (Singh et al., 2024) and the payer preference for the least expensive agent first is unresolved and will intensify as more agents are approved. In regions where advanced therapy is largely unavailable, the priority remains earlier diagnosis, optimal use of conventional therapy and timely surgery.

## 12 Conclusion

The treatment of IBD has changed substantially over the past 5 years. Selective IL-23p19 antagonism has emerged as a highly effective and well-tolerated class with dual indications; upadacitinib has demonstrated among the largest treatment effects of any agent, tempered by class-wide safety warnings that require individualised risk assessment; oral S1P receptor modulators have added convenience at the cost of new monitoring obligations; and subcutaneous formulations and biosimilars have altered the practical and economic delivery of established therapy. Head-to-head trials and living guidelines have begun to replace inference with evidence, and the PROFILE trial has strengthened the case for early advanced therapy in newly diagnosed Crohn’s disease, although its generalisability to ulcerative colitis and to longstanding disease remains untested.

Yet the central limitation is unchanged: no agent achieves remission in more than approximately half of patients in placebo-controlled trials of moderate-to-severe disease, and durable endoscopic healing in fewer still. Higher rates are reported in biologic-naive populations and in responder-enriched randomised-withdrawal maintenance designs, which is itself a reminder that the apparent ceiling is partly a property of trial design. The most promising responses to this limitation are not more potent immunosuppression but better patient selection and rational combination. The anti-TL1A antibodies, with their embedded genetic response biomarker and anti-fibrotic potential, obefazimod, with a mechanism that augments endogenous regulation rather than blocking a single cytokine, and oral IL-23 receptor antagonist peptides, which promise the efficacy of a biologic in tablet form, are the developments most likely to define the next phase. The task for the coming decade is to determine not simply whether these agents work, but for whom.
